# Nanocellulose-Based Materials for Water Pollutant Removal: A Review

**DOI:** 10.3390/ijms25158529

**Published:** 2024-08-05

**Authors:** Hani Nasser Abdelhamid

**Affiliations:** 1 Department of Chemistry, Faculty of Science, Assiut University, Assiut 71516, Egypt; hany.abdelhamid@aun.edu.eg; 2 Egyptian Russian University, Badr City 11829, Cairo, Egypt

**Keywords:** nanocellulose, water treatment, clean water, desalination

## Abstract

Cellulose in the nano regime, defined as nanocellulose, has been intensively used for water treatment. Nanocellulose can be produced in various forms, including colloidal, water redispersible powders, films, membranes, papers, hydrogels/aerogels, and three-dimensional (3D) objects. They were reported for the removal of water contaminants, e.g., heavy metals, dyes, drugs, pesticides, pharmaceuticals, microbial cells, and other pollutants from water systems. This review summarized the recent technologies for water treatment using nanocellulose-based materials. A scientometric analysis of the topic was also included. Cellulose-based materials enable the removal of water contaminants, and salts offer advanced technologies for water desalination. They are widely used as substrates, adsorbents, and catalysts. They were applied for pollutant removal via several methods such as adsorption, filtration, disinfection, coagulation/flocculation, chemical precipitation, sedimentation, filtration (e.g., ultrafiltration (UF), nanofiltration (NF)), electrofiltration (electrodialysis), ion-exchange, chelation, catalysis, and photocatalysis. Processing cellulose into commercial products enables the wide use of nanocellulose-based materials as adsorbents and catalysts.

## 1. Introduction

Water is essential for a living system, representing approximately 71% of the Earth’s surface. The amount of water in the universe is preserved. However, the pace of water consumption has been increasing at more than double the rate of the whole human population. Clean water has become a critical challenge of the 21st century due to the significant rise in water use and issues like water contamination, and the shortage of water. The recent statistics from the World Resources Institute (WRI, https://www.wri.org/freshwater (accessed on 29 July 2024)) reveal that over four billion people face water stress conditions for at least a period of one month per year. WRI also expects that there will be an increase of up to 30% by 2050. It also reported that 1% of the Gross Domestic Product (GDP) could provide water security for all by 2030. There is 25% of the global population living in countries facing water stress. The Agenda 2030 explicitly includes clean water and sanitation as one of the Global Sustainable Development Goals (SDGs #6).

Wastewater treatment is an essential technology for fulfilling the fundamental requirements of human domestic and industrial water. Materials and advanced methods offer high technologies for water treatment. There are several methods for treating wastewater, including precipitation, coagulation, flocculation, complexation, adsorption, filtration (membrane, filter, thin film), ion exchange, Reverse Osmosis, electrodialysis, electrocoagulation, and catalysis (e.g., UV photolysis, photocatalysis, and advanced oxidation processes (AOPs)). Several materials were reported for the water treatment, including natural materials, e.g., bio-based materials [1], and synthetic materials, e.g., metal–organic frameworks (MOFs), polymers, zeolitic imidazolate frameworks (ZIFs) [2], covalent organic frameworks (COFs), metallic nanoparticles, metal oxides, and metal chalcogenides. Among these materials, biopolymers are promising being biodegradable, biocompatible, cheap, available, and can be proceed using recent technologies into custom products.

Nowadays, there is a substantial need to replace products obtained from fossil fuels with alternative materials that are capable of being biodegraded naturally and/or are derived from biological sources. Cellulose is a favorable alternative option for fossil fuel-derived materials. It can also be used for fuel production via biological methods [3]. Cellulose-based materials may solve challenges such as the accumulation of oil reserves, the contamination caused by plastic, and the release of carbon emissions, and offer a sustainable approach. Thus, the production of cellulose is highly important. Several methods were reported for the production of nanocellulose, including mechanical disintegration (e.g., refining and homogenization via homogenizers and microfluidizers), 2,2,6,6-tetramethylpiperidine-*N*-oxyl (TEMPO)-mediated oxidation [4], enzymatic hydrolysis [5], extrusion for mechanical disintegration, periodate–chlorite oxidation, quaternization, sulfonation, and solvent-assisted methods for biochemical pretreatments, and fractionation procedures. Cellulose-based materials have been widely used for biomedicine [6], water treatment [7,8,9], and UV protection [10]. They offer sustainable purification technologies [11]. They are effective materials for membrane-based technologies with and without substrates [12].

This review examines the considerable potential of nanocellulose (NC) for eliminating contaminants in wastewater treatment. We explore the distinctive characteristics of NC, such as its elevated aspect ratio, extensive surface area, and functional groups, including hydroxyl and carboxylic groups, which make it an exceptionally efficient adsorbent for diverse contaminants. This review assesses the effectiveness of NC in removing pollutants such as heavy metals, dyes, oils, and drugs from wastewater. We examine the applications of NC in both adsorption and membrane filtration, emphasizing its benefits, such as biodegradability, renewability, and cost-effectiveness in comparison to conventional techniques. In addition, this paper analyzes the progress of composites based on NC, in which NC is coupled with other materials to augment its ability to remove contaminants. We discuss the present obstacles and prospects for research, with a focus on the importance of improving the production processes of NC and investigating its potential for large-scale use in practical wastewater treatment applications.

**Figure 1:**
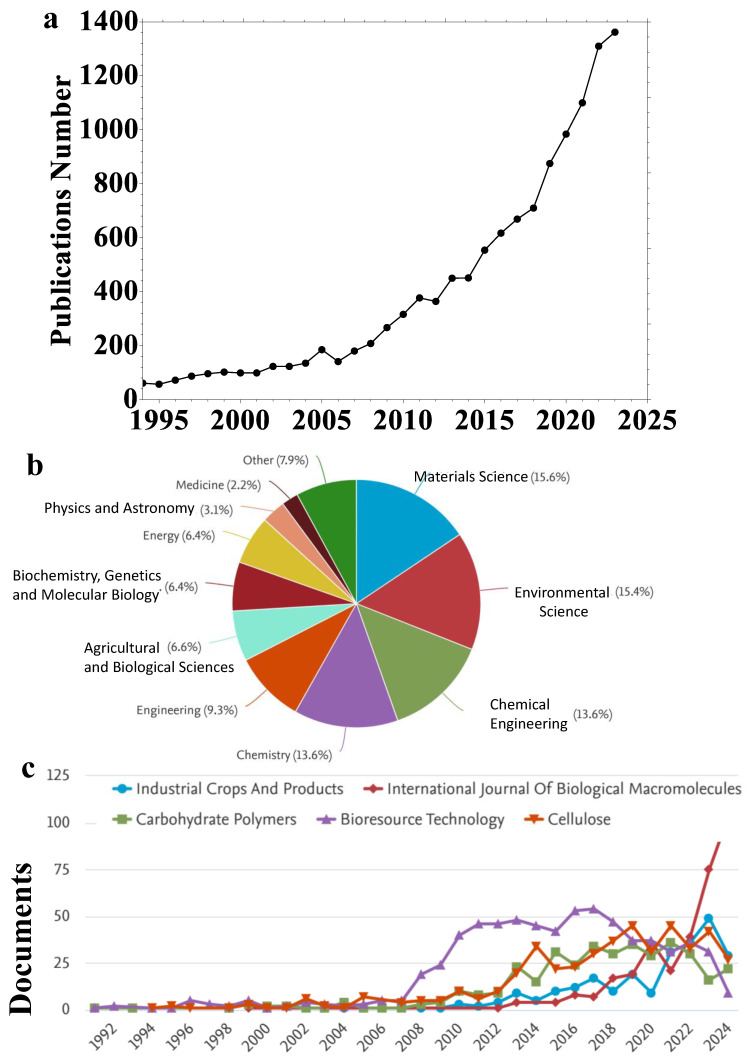
Scientometric analysis of cellulose based on (**a**) publications number, (**b**) subjects, and (**c**) sources. Data analysis was performed using the Scopus database for the words ‘cellulose AND water treatment’.

Figure 1 shows the scientometric analysis of cellulose applications in water treatment. There are exponential increments in the publications number over the year (Figure 1a). Cellulose was applied to several subjects, including materials science, environmental science, chemical engineering, chemistry, engineering, energy, and others (Figure 1b). The topic of cellulose and its applications in water treatment can be followed in several good journals, as shown in Figure 1c.

## 2. Chemical Structure of Cellulose

**Figure 2:**
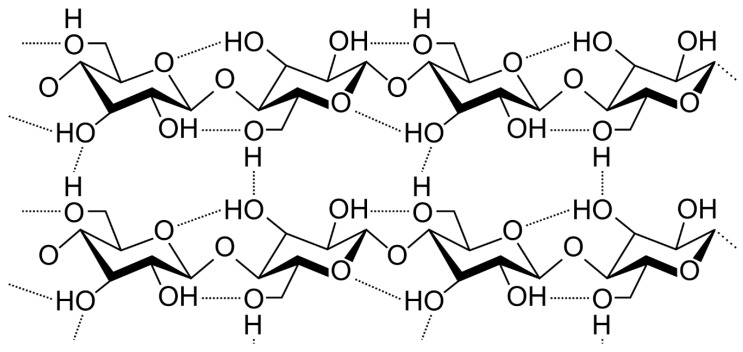
Chemical structure of cellulose and their hydrogen bonds.

Cellulose products have been crucial in the documentation and dissemination of human civilization from the time of the Egyptian papyri. Cellulose as a chemical raw material has been used for around 150 years (Figure 2). Cellulose is often regarded as the highest abundant polymer on Earth, comprising around 40–50% of the biomass. It is mostly conserved inside plants and microorganisms in their native environments. It can be generated in nature by photosynthesis. It has long been seen as a plentiful and sustainable green resource.

Anselme Payen, a French scientist, made the initial discovery of cellulose in 1838 [13]. Originally, he employed the term “Cellulose” to denote the processed plant material that is now widely recognized as plant pulp. He asserted that cellulose exhibits a higher degree of compaction in comparison to starch. In addition, he stated that specific treatments have the potential to modify the consistency of cellulose. The enzymatic conversion was used to explore the differences in properties between cellulose and starch, which were related to the isomeric forms of both substances. In 1891, Schulze utilized the term “cellulose” in his research [14]. Hemicelluloses were coined by the German chemist Ernst Schulze in 1891 to refer to polysaccharides found in plant cells, which are composed of galactose, mannose, arabinose, or xylose. In 1813, the Swiss botanist Augustin Pyramus de Candolle introduced the term “lignin” to refer to the non-hydrolyzable component of wood. This term was later adopted by Franz Ferdinand Schulze in 1856 [14].

The determination of the cellulose structure may be attributed to the research conducted by Staudinger in 1920. These discoveries, in addition to Staudinger’s investigations, signified the identification of the polymeric state of these materials. Sponslor and Dore (1926) made notable contributions to the progress of the structural model of cellulose. The proposed original model was a chain model that depended on glucopyranose [15]. Scientists have determined that cellulose is composed of fibers in the nano or micro regimes that are present in wood after conducting thorough research and applying it in various industries. These achievements were the birth of polymer science.

Cellulose is a pure semicrystalline organic polymer that consists of both ordered (crystalline) and disordered (amorphous) regions within the microfibril. The inherent level of crystallinity often falls between the 40–70% range and is contingent upon the source of cellulose and the technique used for separation. Cellulose consists of a straight long chain formed of β-D-glucopyranose (glucose) molecules, which are joined together by 1,4-glycosidic bonds (Figure 2). It consists of a reducing end, a non-reducing end, and internal rings of thousands of glucose molecules. The structure of this chain exhibits a chair conformation. The hydroxyl groups in cellulose indicate its highly cohesive microfiber network structure, which is created by strong hydrogen bonds involving polyhydroxy groups. The hydrogen bonding phenomenon also enhances the cellulose’s ability to withstand dissolving in typical solvents.

Hemicellulose (HC) is the second most prevalent polysaccharide, comprising around 20–35% of lignocellulosic biomass. It is a complex, heterogenous, and branching polysaccharide. It consists of many carbohydrate repeating units, including glucose, xylose, mannose, arabinose, galactose, and others. The proportion of hemicellulose changes significantly depending on the source of lignocellulosic biomass. Due to its diverse sources, low molecular weight, and variable chemical composition, the commercialization potential of HC has not been adequately explored for value-added applications, unlike other natural polysaccharides.

The molecular size of cellulose can be determined using several methods, such as size exclusion chromatography (SEC). The SCE method evaluates the molecular weight distribution of dissolving pulp samples. The cellulose pulp is usually dissolved in a solvent, such as a mixture of dimethylacetamide (DMAc) and lithium chloride (LiCl). The water in the cellulose suspensions was extracted by solvent exchange using methanol and DMAc and then dissolved in DMAc/LiCl. The analysis of the molecular weight can be performed using pullulan for calibration purposes and a refractive index as a detector.

The degree of polymerization (DP) varies depending on the origin of cellulose. The DP values for natural wood, cotton, and Valonia are 10,000, 20,000, and 44,000, respectively. The outcome might also vary based on the methodology used for segregation and refinement. As an example, DP may range from 200 to 500 in regenerated cellulose and reach 1000 in bleached kraft pulps. DP can be evaluated using several empirical equations using intrinsic viscosity, *η* (mL/g).

Cellulose has several functional groups, such as hydroxyl groups. Cellulose’s functional groups enabled hydrophilic properties [16]. The surface functional groups of cellulose enable modification with several molecules [17]. They can also be used to process the materials into aerogels and hydrogels. Cellulose hydrogels can be modified via physical (e.g., blending, addition of fillers, and incorporation) or chemical methods (e.g., crosslinking and grafting) [18].

The process of cationization of cellulose fibers entails introducing positive and negative charges to the cellulose employing electrophilic addition, affinity, or nucleus addition, which involves the introduction of certain groups or components to enhance the swelling of cellulose in water. Alternatively, the insertion of an identical charge may be used to induce repulsion between the fibers, therefore diminishing the cohesiveness between them. Cellulose pretreatment improves the process of breaking down cellulose into smaller fibrils while also introducing modified structures or groups with specific functions onto the surface of the cellulose. Consequently, the interactions among different components in the experiment create challenges in maintaining control, leading to subpar consistency in the cellulose nanofibers (CNFs).

Cellulose exhibits good properties, including unique optical characteristic features. In 1959, Marchessault et al. observed that nanocrystal suspensions exhibit birefringence after they reach a certain concentration threshold [19]. In 1992, Revol et al. revealed that nanocrystal cellulose (NCCs), formed by sulfuric acid hydrolysis, really exhibits a chiral nematic liquid-crystalline phase [20]. After this finding, much research focused on investigating the optical and liquid-crystalline characteristics of cellulose suspensions. They reported the orientation of crystals when subjected to an external magnetic field [21] and a rotating magnetic field [22].

## 3. Nanotechnology and Nanocellulose

Nanotechnology, technology for particle sizes less than 200 nm, has made significant progress in various areas, such as water treatment [23,24,25,26]. Nanomaterials can be utilized in various techniques, including chemical precipitation, ion exchange, adsorption, membrane separation, filtration, coagulation/flocculation, flotation, catalysis, and electrochemical-based processes. Cellulose-based materials show great promise among the vast array of nanomaterials. Cellulose possesses numerous benefits as it is a plentiful and non-toxic compound. The presence of functional groups in cellulose allows for systematic chemical modification using various methods and reagents [27]. It can be produced using additional biopolymers like chitosan [28,29] and inorganic nanoparticles [30,31,32], such as metal oxides [32]. It facilitates the conversion of materials into marketable products, such as membranes [29], using sophisticated engineering processes [33].

Cellulose can be produced in a nano-regime, denoted as nanocellulose (NC). There are several derivatives of nanocellulose, including cellulose nanofibrils (CNFs), cellulose nanocrystals (CNCs), bacterial nanocellulose (BNC), enzymatic nanocellulose (ENC), and TEMPO-oxidized cellulose nanofibrils (TOCNFs). The differences among these derivatives are summarized in Table 1 based on their size. The properties of the materials depend on several parameters, including raw material sources, production methods, treatments, and modifications.

## 4. Production Methods for Nanocellulose

Cellulose may be obtained from several sources, including hardwood and softwood from trees [34], fiber from seeds (e.g., cotton and coir), fibers from plants (e.g., flax, hemp, jute, and ramie), grasses (e.g., bagasse and bamboo), and marine organisms including tunicates, algae, fungi, crustaceans, and bacteria. Wood consists of around 40–50% cellulose by weight, with half of it being in nanocrystalline form and the other half in amorphous form. Plant cellulose is mostly located inside the cellular structures of plant fibers, namely in the cell walls. The formation of the cell wall occurs in a specific order, leading to varied chemical compositions and architectures.

The cellulose raw materials used for the extraction of CNFs may be obtained from several sources, including wood of trees, plant seed fibers (e.g., cotton fiber or lint, and kapok), plant bast fibers (e.g., hemp, flax, and abaca), and other herbs (e.g., bagasse, straw, and bamboo fiber). The average polymerization degree of wood cellulose is around 10,000, but that of cotton fiber is notably greater, at approximately 15,000. Conversely, the polymerization degree of herbal cellulose is lower. Bast fiber and herbaceous straw have fiber lengths of 120–180 mm and 1–2 mm, respectively. The wood fiber length varies between about 3 and 5 mm. Seed fibers, like cotton, often have a cellulose content above 95%, which is the highest among all types of fibers. Wood fibers, on the other hand, include extra impurities such as lignin and ash. Softwoods possess a higher concentration of fibers and longer individual fiber strands in comparison to hardwoods. Cellulose is mostly present in fully developed plant cells inside unprocessed plant parts. Thick-walled plant cells, also known as fibers, are often used in the production process to reduce the occurrence of fiber fragments in CNF products. Generally, when the proportion of raw fiber materials used to produce CNFs is increased, it leads to the formation of thinner and more uniform individual fibers, decreased presence of random cells, and enhanced strength and fibrillation degree of the CNFs. As a result, nanofibers of exceptional quality are mostly made up of 100% cotton fibers.

In 1870, the Hyatt Manufacturing Company conducted a procedure that included the reaction of cellulose with nitric acid to produce cellulose nitrate, which served as the foundation for the creation of celluloid, the first thermoplastic polymer material. The purification of cellulose from plant fibers entails chemical procedures such as alkali extraction and bleaching. There are several studies on the extraction of cellulose fibrils from plant fibers, but some unsolved challenges persist. The majority of the current techniques focus on cellulose extraction without substantial degradation. A further concern emerges from the scattering of cellulose fibrils inside polymer matrices, especially in situations where they display hydrophobic characteristics.

Nanocellulose refers to cellulose particles with dimensions ranging from 1 to 100 nm. It is produced from a natural cellulose polymer by a top-down method that includes mechanical or chemical breakdown, thanks to its structure and semicrystalline characteristics. It may be classified into three categories according to the manufacturing circumstances, which impact the dimensions, content, and qualities. The first classification comprises cellulose nanocrystals (CNC), nanocrystalline cellulose (NCC), cellulose nanowhisker (CNW), or cellulose whiskers. The second group is cellulose nanofibrils (CNFs), which are also reported under other names such as nanofibrillated cellulose (NFC), microfibrillated cellulose (MFC), cellulose nanofibers, or nanofibrillated cellulose. The third class of nanocellulose can be bacterial cellulose (BC). To provide uniformity across the many types of nanocellulose, several technical committees and organizations, including ISO TC 6 and TC 229, TAPPI, and CSA Z5100-14, were reported. This is necessary because there is a lack of consistency in the terminology used to describe cellulose nanoparticles. Nanocellulose may be classified into two main categories: (1) CNC, and (2) CNFs. CNCs are created by acid treatment. While CNFs are mostly generated via mechanical disintegration.

### 4.1. Acid Hydrolysis

The process of defibrillation or breaking down cellulose with an acid, known as acid hydrolysis, was first documented in 1949 [35]. The acid hydrolysis of cellulose fibers in water was used to produce CNC [35]. Acid hydrolysis involves the infiltration of hydronium ions into the less structured portions of cellulose chains. This infiltration helps break down the glycosidic connections, leading to the release of distinct crystallites when mechanical treatment, such as sonication, is applied. Several strong acids have shown successful decomposition of cellulose fibers, with hydrochloric and sulfuric acids being used for this objective. Furthermore, the use of phosphoric acid (H_3_PO_4_), hydrobromic acid (HBr), and nitric acid (HNO_3_) has been recorded in the manufacturing process of crystalline cellulose nanoparticles. Sulfuric acid is often used as a hydrolyzing agent due to its ability to react with surface hydroxyl groups via an esterification process, facilitating the grafting of anionic sulfate ester groups.

Concentrated sulfuric acid is often used to degrade the amorphous components of cellulose while maintaining the integrity of the crystalline segments. This process yields rod-shaped CNCs that possess sulfate functional groups on their surface. CNCs are often produced with a diameter that falls within a range from 3 to 35 nm and a length that ranges from 200 to 500 nm.

Acid hydrolysis may greatly reduce the molecular weight of cellulose, leading to the formation of short, fibrous crystallites known as microcrystalline cellulose (MCC). Therefore, the reinforcing effect may be diminished as a result of acid hydrolysis. Challenges of acid hydrolysis can be solved using applied alkali treatment or the use of a post-treatment procedure.

### 4.2. Mechanical Method

Mechanical methods can be used to disintegrate or delamination of cellulose fibers into nanocellulose. It can include several configurations: high-pressure homogenizer, microfluidizers, supergrinding, refiner-type treatments, combinations of beating/rubbing/homogenization, high-shear refining, and cryogenic crushing. The mechanical method starts at ambient temperature (20–25 °C). Then, the temperature increases to 60–70 °C. A study reported that raising the temperature from 20 °C to 70–80 °C promotes homogeneity [36].

CNFs were extracted from eucalyptus fiber in 1982 using a high-pressure homogenizer. The approach included passing a diluted mixture of cellulosic wood pulp fibers and water through a mechanical homogenizer, which creates microfibrillation by inducing a significant increase in pressure. The application of intense shearing forces during the treatment leads to the formation of densely intertwined networks of nanofibrils, including both crystalline and amorphous regions. These objects have a large ratio of length to width and may create gels in water that show a decrease in viscosity under shear stress and display the property of becoming less viscous over time. Cellulose fibers may be degraded under certain processing conditions to produce CNFs. The cross-sectional dimensions of these CNFs range from around 5 nm, which are classified as elementary fibrils, to tens of nanometers, which correspond to individual microfibrils and their bundles. CNFs typically have a diameter that falls within a range from 5 to 50 nanometers and a length of a few micrometers. The synthesized CNFs exhibited notable properties, such as good biocompatibility, extraordinary mechanical properties, and excellent transparency. Unlike other types of nanocellulose fibers, CNFs can be easily produced. This approach has been the main focus of the industrial production of nanocellulose.

MFC is often produced by subjecting wood pulps to high-pressure homogenization. Pulp is obtained by the chemical processing of wood. Sodium hydroxide and sodium sulfide are combined to generate kraft pulp, which mostly comprises cellulose fibers. Sulfite pulp is the term used to describe cellulose fibers that are produced by the pulping process using salts of sulfurous acid [37]. This kind of pulp has a greater quantity of by-products inside the cellulose fibers. The delamination process can be expedited by the incorporation of hydrophilic polymers, such as carboxymethyl cellulose (CMC) or hydroxypropyl cellulose (HPC). These polymers decreased the probability of blockage and enabled the use of higher pulp concentrations during homogenization. Usually, MFC has to undergo the homogenization process 5–10 times to obtain a gel-like texture. As a result, the energy usage is considerably high.

To facilitate the separation of nanofibrils during the mechanical disintegration process, several methods have developed as additional stages to create nanocellulose through the investigation of several pretreatment techniques, such as chemical treatments, the nitro-oxidation method [38], TEMPO-mediated oxidation, carboxymethylation [39], carboxylation [38], sulfonation [40], quaternization [41], solvent-assisted pretreatments [42] including ionic liquid [43], acid hydrolysis, or enzymatic hydrolysis. These methods were applied to enhance mechanical disintegration.

CNF has gained increased attractiveness as a material for commercial applications. However, continuous research is focused on improving the effectiveness of existing procedures and developing advanced methods that might enhance the production process or impart new properties to the CNF. The addition of charged groups to the pulp fibers has been well-recognized as a means to improve the separation of the fiber walls. By introducing carboxymethyl groups, a completely separated carboxymethylated MFC can be achieved. To maximize pulp swelling, these groups should exist in the form of their sodium salts. Pulps that are more swollen have less cell wall cohesiveness compared with less swollen pulps, making them more susceptible to delamination. Therefore, holocellulose pulps (a type of wood pulp that contains primarily cellulose), which consist of anionic polysaccharides, are more susceptible to delamination. Persulfate-oxidized cellulose was reported as a suitable cellulose precursor for MFC production.

The mechanical method suffers several challenges. It included passing a diluted mixture of cellulosic wood pulp fibers and water through a mechanical homogenizer. When a solution of cellulosic pulp fibers is homogenized, the technique is often repeated many times to enhance the level of fibrillation. A study reported that the disintegration was improved as the number of passes through the homogenizer increased, eventually reaching a total of 30 passes at a high pressure of 100 MPa [44]. Currently, a more effective method is used to disintegrate MFC nanofibers. This procedure included using TEMPO oxidation as a preliminary step before mechanical treatment [4]. An increased number of passes directly correlates with an augmented energy need for disintegration. To promote disintegration, one may decrease the length of the fiber by mechanical cutting or weaken the fiber cell wall by subjecting it to acid hydrolysis before homogenization. Thus, concentrated solutions require several steps. The disintegrated cellulose requires further steps to increase the concentration. Functional groups, such as carboxyl or carbonyl, are often introduced into wood- or plant-derived cellulose during the production process. The use of several mechanical shearing steps enables the liberation of varying numbers of individual microfibrils [45].

A study also documented the use of an enzyme-assisted mechanical technique to break down MFC [46]. This technique involves the use of endoglucanases or acid hydrolysis, together with mechanical shearing, to disintegrate MFC present in the cell wall of cellulosic wood fiber pulps. Enzymes were used to enhance the breakdown process, leading to MFC nanofibers with increased average molar mass and higher aspect ratio in comparison with nanofibers produced with acidic pretreatment [46]. The enzyme-assisted mechanical technique offers strong reinforcement in polymer nanocomposites [46].

### 4.3. Biological Methods

Enzymatic disintegration and bacteria were used for the production of nanocellulose. Cellulose is resistant to breakdown by specific enzymes. Aerobic fungi, such as *Trichoderma*, *Phanerochaete*, and *Aspergillus* species, may decompose cellulose by releasing a mixture of hydrolytic enzymes that act autonomously yet collaborate. A cellulase enzyme set may have at least seven unique enzymes derived from different protein families. Nevertheless, a minimum of four distinct functional types of cellulases may be distinguished: cellobiohydrolases, referred to as A- and B-type cellulases, can degrade cellulose with a high degree of crystallinity. Conversely, endoglucanases, also known as C- and D-type cellulases, often need a certain degree of structural instability to degrade cellulose. The cooperative activity of cellobiohydrolases and endoglucanases produces substantial synergistic outcomes [47]. A pure form of C-type endoglucanase was reported as an environmentally sustainable pretreatment of wood pulp fibers that have a high cellulose content [46]. The goal was to improve the breakdown of MFC nanofibers.

Bacterial cellulose (BC) is a kind of cellulose that is synthesized by bacteria in a liquid growth media containing a source of sugar [48]. The length of this operation ranges from a few days to a maximum of two weeks. Both BC and plant cellulose have similar chemical structures. However, it does not possess any functional groups other than hydroxyl. BC lacks additional polymers, such as lignin, hemicelluloses, or pectin, which makes it appropriate for biomedical applications. BC is produced in the form of helical strips that have dimensions ranging from 3 to 4 nm in width and 70 to 140 nm in length, with a length that is more than 2 µm. The polymer has a polymerization degree that spans from 3000 to 9000 and demonstrates a crystallinity level between 80 and 90%.

Bacterial nanocellulose (BNC) is synthesized by aerobic bacteria, namely acetic acid bacteria belonging to the Gluconacetobacter species, as a unique component of their biofilms. These bacteria are found everywhere in nature and play a role in breaking down sugars and plant polysaccharides via fermentation. The bacteria are cultivated in conventional liquid nutrient solutions, where BNC is produced as a polysaccharide at the boundary between the air and liquid phases. BNC is created using biotechnological assembly methods employing low-molecular-weight carbon sources, namely D-glucose. This can be considered to bottom-up approach compared with a top-down procedure, e.g., acid and mechanical approaches. The BNC hydrogel consists of a nanofiber network with a diameter ranging from 20 to 100 nm, and it contains a maximum of 99% water. It has a high level of cellulose purity, together with a substantial weight-average molecular weight (Mwt.), a high degree of crystallinity, and exceptional mechanical stability. The fabrication approach enables the synthesis of cellulose by fermentation, namely in the domain of white biotechnology. Additionally, it allows for the manipulation of the morphology of the cellulose entities and the arrangement of the nanofiber network throughout the synthesizing process.

## 5. Nanocellulose-Based Composites

The functional groups of cellulose can be modified via esterification, halogenation, etherification, sulfonation, acetylation, silylation, amination, sulfonation, phosphorylation, and oxidation. The modification enables new functional groups for water removal [49]. Cellulose can also be combined with other materials via mixing or grinding to form homogenous or heterogeneous materials. It can be blended with other materials, including natural (e.g., biopolymers and clays) or synthetic materials (polymers, metallic nanoparticles, metal oxides, two-dimensional (2D) nanoparticles, and metal–organic frameworks (MOFs)).

Cellulose/biopolymer composite was reported [50]. Chitosan/cellulose composite was reported [51]. Biopolymers such as chitosan improved cellulose’s properties such as hydrophilicity, non-toxicity, biodegradability, biocompatibility, and eco-friendliness. These properties enabled the high affordability of cellulose-based materials as suitable adsorbents. Biopolymers enabled several extra functional groups that improved interaction with other nanomaterials. The functional groups of the composite can be used to extract pollutants via several interactions, including hydrogen bonding and hydrophobic interactions. Biopolymers such as alginate enable the processing of cellulose into beads [52].

Nanocomposite of biopolymers containing cellulose for certain materials may show drawbacks such as limited surface area and poor adsorption capacity. Biopolymers increase the swelling of cellulose materials when exposed to water. They also show a varying degree of shrinkage or expansion in reaction to other solvents, such as organic solvents and salt solutions. They caused enlargement that may diminish the mechanical robustness and coherence of cellulose materials.

**Figure 3:**
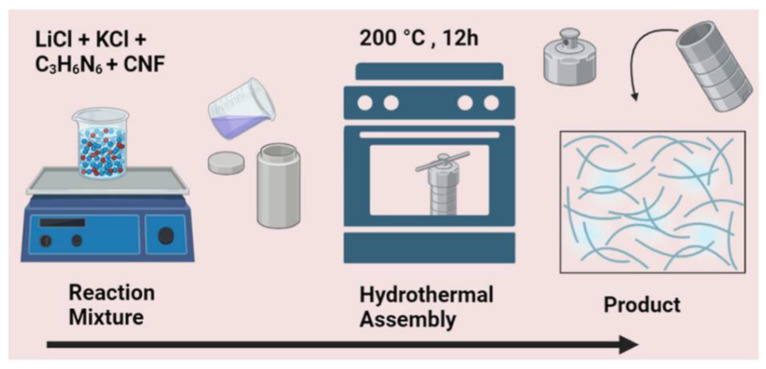
Schematic representation of the preparation of the CNF@C_3_N_4_ sorbent [54]. Copyright© 2023 the authors. Published by The American Chemical Society. This publication is licensed under CC-BY-NC-ND 4.0.

Cellulose can be combined with other nanoparticles, e.g., graphene oxide [53], graphitic carbon nitride (C_3_N_4_) [54], MOFs [55,56], and zeolitic imidazolate frameworks (ZIFs) [57,58,59]. The synthesis of these composites depends on the type of the materials combined with cellulose and also on the intended applications. For example, C_3_N_4_ nanosheets were produced on cellulose nanofiber surfaces using an environmentally benign salt melt method (Figure 3) [54].

## 6. Applications of Nanocellulose for Pollutant Removal

There are several methods for the removal of water pollutants. Water treatment can involve a single process or multiple processes. Chemical precipitation is a traditional technique used to eliminate metal ions and anionic species. This technique entails the introduction of chemical reagents that interact with impurities and generate a solid substance that settles out of the solution. Therefore, the contaminants after precipitation can be eliminated through the process of decantation or filtration using a filter paper or membrane. Previous studies have identified cellulose as one of the precipitating agents used in the co-precipitation of metal ions, specifically Cd^2+^ ions [21] and Cu^2+^ ions [60]. The precipitates are typically filtered using a commercially available cellulose Whatman^®^ filter paper.

Coagulation or flocculation is a highly effective technique for water treatment. This technique is based on the introduction of a chemical coagulant, which leads to the neutralization of charged particles in water. As a result, the particles come together and settle down. The process involves the introduction of reagents, such as cellulose, aluminum chloride (AlCl_3_), and ferric chloride (FeCl_3_), which induce the coagulation of contaminants. This technique is applicable for the elimination of metals, suspended particles, and total dissolved solids (TDSs). Nevertheless, its capability is restricted to the creation of a substantial quantity of alkaline sludge. The coagulant has the potential to generate secondary contaminants. Therefore, the utilization of biopolymers, such as cellulose, as a coagulant is interesting [61,62].

Cellulose is a sustainable biomaterial that can be processed with minimal steps [63]. A study documented the use of CNC produced from sawdust as a coagulant for Ni^2+^ and Cd^2+^ ions [64]. The adsorption capabilities for Ni^2+^ and Cd^2+^ ions were 956.6 mg/g and 2207 mg/g, respectively [64]. When treated with hexadecyltrimethylammonium bromide (HDTMA-Br), the performance of the substance is enhanced compared to a commercially available coagulant like R2T2 [63].

The adsorption process relies on the immobilization of pollutants (adsorbate) on the surface of solid materials (adsorbent). This process is widely used for the elimination of contaminants, e.g., metal ions and dyes. The interactions can occur through two types of forces: (1) physical forces (e.g., electrostatic interaction, hydrogen bonds, and van der Waals forces) and (2) chemical forces (e.g., ion exchange or complexation). The process of adsorption has various benefits, including a high level of reversibility, which allows for the reuse of the adsorbent material utilizing desorption. Therefore, the pollutants can be repurposed and reevaluated as a viable source for chemical compounds. Adsorbents that possess a substantial surface area, porosity, and tiny particle size typically exhibit significant levels of adsorption efficiency.

The adsorption of heavy metal ions on cellulose adsorbent can be achieved via ion-exchange materials, such as cellulose, depending on the replacement of heavy metal ions with harmless ions like sodium (Na^+^) or potassium (K^+^). This approach is exclusively employed for metal ions. Therefore, it is incapable of eliminating additional contaminants such as dyes or microorganisms. Moreover, the elevated levels of metal ions, such as sodium (Na^+^) and potassium (K^+^), in drinking water might have adverse effects on the ecological system and lead to neurological disorders.

The separation of a mixture containing multiple liquids can be achieved through various methods, including funnel separation (which involves separating two liquids that do not mix), distillation (which relies on the evaporation of water into a vapor at different temperatures based on their boiling points), and pervaporation (a technique for separating liquid mixtures by partially vaporizing them through a membrane). A study described the use of Zeolite 13X/regenerated cellulosic membrane for separating a mixture of water–glycerol through pervaporation [65]. The membrane exhibited a flow of 65 g/m^2^·h and a selectivity of 1681 for a 90 wt.% glycerol aqueous solution. A cellulose membrane containing 20 wt.% of Zeolite 13X exhibited superior selectivity in comparison to cellophane or the original cellulosic membrane [65].

Organic contaminants can be eliminated using biological techniques. These methods are extensively employed on a large industrial level. They rely on the utilization of biological species, e.g., bacteria, that can decompose organic molecules. These processes can be categorized into aerobic (in the presence of oxygen) and anaerobic (in the absence of oxygen) activities. The technique is efficient in eliminating organic contaminants. Nevertheless, it is typically necessary to employ an additional treatment method, such as dissolved air flotation (DAF), to eliminate any residual substances that remain after the initial treatment, such as sedimentation. The process necessitates a significant amount of energy. Advanced technologies like the membrane aerated biofilm reactor (MABR) can save up to 90% of the energy needed for the aeration process, which is promising for the future.

The advanced oxidation process (AOP) has gained recognition as a prominent method for water treatment. They exhibit exceptional efficiency in eliminating contaminants that are derived from organic pollutants. The mechanism of these methods is based on the decomposition of organic pollutants into smaller fragments that have reduced detrimental effects. They may be utilized without generating any more garbage. The degradation process can occur through several mechanisms, such as photocatalysis reaction (using a catalyst and light source), pyrolysis (using heat), ozonation (using ozone, O_3_), or ionization (using electrons).

Nanocellulose-based materials are good adsorbents for water contaminants. Water pollutants can be removed via enrichment in a surface of materials such as nanocellulose [66,67]. This process is defined as adsorption and can be performed via physical and chemical interactions, leading to physisorption and chemosorption, respectively. The adsorption process can be performed via ion exchange or chemical complexation between pollutants and cellulose functional groups.

Adsorption, in general, can be explained using several models used to understand how pollutants stick to adsorbent materials. These models are Langmuir, Freundlich, Dubinin–Radushkevich (D-R), Tampkin, Flory–Guggins, Hill, Frenkel–Halsey–Hill, Redlich–Peterson, Sips, Toth, Koble–Corrigan, Khan, Radke–Prausnitz, and MacMillan–Teller. Few of these methods are the common models for adsorption. These models rely on various assumptions. For example, Langmuir assumes a single layer of pollutant molecules on a uniform surface with a limited number of binding sites. Freundlich is better suited for uneven surfaces where pollutants can form multiple layers. D-R helps determine the type of adsorption (physical or chemical) and the energy involved, considering a varied surface. Temkin assumes a gradual decrease in the energy released during adsorption. The Flory–Huggins model indicates how favorable and spontaneous the adsorption process is. Hill considers how binding sites on a large molecule can influence each other. Redlich–Peterson (R-P) combines Langmuir and Freundlich, working across various concentrations and surface types. The sip model is useful for predicting adsorption on uneven surfaces at different pollutant concentrations. It behaves similarly to Freundlich at low concentrations and Langmuir at high concentrations.

**Figure 4:**
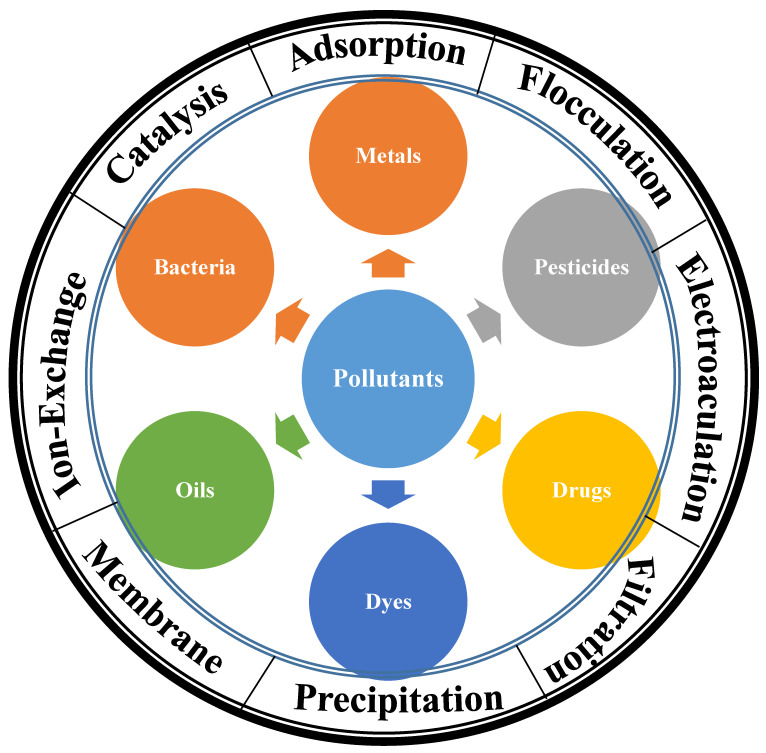
Nanocellulose applications for removal of water pollutants.

Nanocellulose is a suitable adsorbent for the removal of several pollutants [68], as shown in Table 2, including heavy metal ions [69,70], oils, and dyes [71]. The following sections summarize the removal of water pollutants via several procedures, including adsorption, coagulation, and catalysis (Figure 4).

### 6.1. Removal of Heavy Metal Ions

Heavy metals are hazardous and non-biodegradable pollutants that are commonly found in wastewater. Various methods can be employed to eliminate heavy metal ions from wastewater, including adsorption, precipitation/sedimentation, coagulation/flocculation, and electrochemical treatment (such as electro-precipitation). Out of these technologies, the adsorption process shows promise because it is inexpensive and highly effective at removing substances, and the metals that are adsorbed can be utilized later if necessary. Cellulose-based materials have been shown as effective for adsorbing various metal ions, including radioactive (e.g., UO_2_^2+^ [72]), carcinogenic (e.g., As^3+^) [73,74], bioinorganic (e.g., Cu^2+^ [75]), and toxic metal ions (e.g., Cd^2+^ [76], Cr^6+^ [77], Pb^2+^ [78,79], and Hg^2+^ [80]), see Table 2. The functional groups present in cellulose materials have a significant impact on the adsorption of metal ions. TOCNF exhibited greater affinity for Cu^2+^ ions and dye molecules in comparison with CNC [81]. Similarly, it was noted that carboxylated CNCs had a greater capacity for metal adsorption compared to non-modified CNFs, which were produced through mechanical treatment [82]. Cellulose nanofibers (CNFs) were treated with CMC (referred to as CNF-CMC) to enhance their ability to adsorb Ag^+^, Cu^2+^, Pb^2+^, and Hg^2+^ ions. This modification was performed because CNF contains a significant amount of negatively charged groups, such as hydroxyl and carboxyl groups [83]. Cellulose materials, like MFC, can undergo grafting with methyl and phosphate functional groups to enhance their capacity for adsorbing metal ions [84]. The O-SO_3_^−^ functional group, which contained CNC and was changed with sulfuric acid during hydrolysis, showed a greater capacity for adsorbing Ag^+^ ions (34 mg/g) compared with CNF, which was produced through mechanical grinding (14 mg/g) [85]. Succinic anhydride was used to modify the hydroxyl group of CNC, resulting in SCNCs. This modification was done to enhance the adsorption capacity of SCNCs for Pb^2+^ and Cd^2+^ ions [86]. Sodium cyanide can be further changed by adding sodium bicarbonate (NaHCO_3_) to make sodium cyanide salt, known as NaSCNCs. The sodium adsorbent, specifically NaSCNCs, exhibited greater adsorption capabilities in comparison to SCNCs.

**Figure 5:**
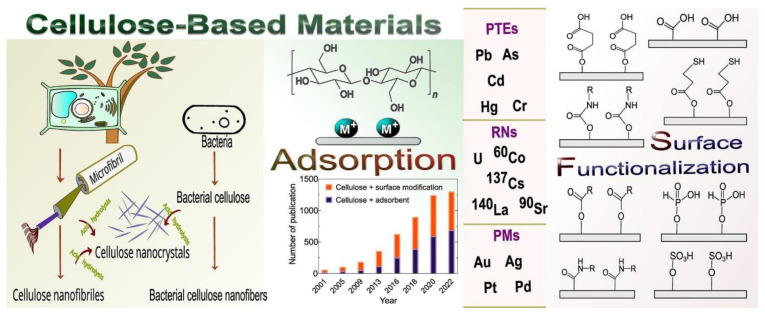
Removal of heavy metals, including PTEs, potentially toxic elements; RNs, radionuclides; PMs, precious metals. Figure reprinted from Ref. [87] Copyright© 2024 Elsevier B.V.

The surface of cellulose can be modified with a wide number of functional groups that enable good adsorption of heavy metal ions via mechanisms such as complexation and coordination (Figure 5) [87]. Carboxycellulose nanocelluloses were produced from untreated Australian spinifex grass using a nitro-oxidation method involving nitric acid and sodium nitrite [38]. It was denoted as nitro-oxidized cellulose nanofiber, NOCNF. The synthesized nanocelluloses have low crystallinity (50%), high surface charge (−68 mV), and hydrophilicity (static contact angle 38°). The NOCNF was discovered to be an efficient medium for the removal of Cd^2+^ ions from water. A dilute NOCNF suspension (0.20 wt.%) effectively eliminated Cd^2+^ ions throughout a wide concentration range from 50 to 5000 parts per million (ppm) within a short time of 5 min. According to the Langmuir isotherm model, the NOCNF material has a maximum capacity to remove Cd^2+^ ions of around 2550 mg/g. The NOCNF demonstrated the greatest level of removal effectiveness, reaching 84% at a Cd^2+^ concentration of 250 ppm. NOCNF nanomaterials are efficient in removing Cd^2+^ ions from water [38].

An adsorbent consisting of ion-imprinted polymer (MIP)-grafted nanocellulose (NC) was developed to address the significant risk posed by Pb^2+^ and Hg^2+^ in wastewater [88]. The study used medical cotton wool as the primary material to produce a nanocellulose suspension using acid-catalyzed hydrolysis using a mixture of sulfuric and hydrochloric acid. Carbonyl diimidazole (CDI) was used for its high reactivity to combine with acrylic acid (AA) to produce reactive intermediates. These intermediates then interacted with nanocellulose to AA-CDI-NC. To improve selectivity toward Pb^2+^ and Hg^2+^, the materials were modified with crown ether and thymine, respectively. The adsorption process was interpreted following the secondary kinetic model and the Langmuir adsorption isotherm model [88].

Biopolymers/cellulose composites were intensively used for water treatment [1]. Rice wastes and polyethersulfone (PES) were used as starting materials for cellulose (CE) and its composite with sulfonated polyethersulfone (CE-SPES) [89]. The materials have been used for the extraction of Zn^2+^ ions from wastewater. The presence of a negative change of enthalpy (ΔH°) and change of entropy (ΔS°) values indicates that the adsorption of Zn^2+^ results in the release of heat and the arrangement of Zn^2+^ ions in a more ordered fashion on the adsorbent’s surface. The negative free energy (ΔG°) and change of enthalpy (ΔH°) results indicate that the adsorption of Zn^2+^ is spontaneous and releases heat. Negative ΔS values suggest a high level of organization of Zn^2+^ ions on the surface of adsorbents. In summary, the results suggest that CE-SPES, with an adsorption energy of 0.86 kJ/mol, is superior to CE, which has an adsorption energy of 0.45 kJ/mol, in removing Zn^2+^ from liquid waste [89].

Nanocellulose/2D nanomaterials were used for heavy metal adsorption [90]. The synthesized material of CNF@C_3_N_4_ exhibited selective adsorption of Ni^2+^ and Cu^2+^ ions from samples of electroplating effluent [54]. Fixating g-C_3_N_4_ onto solid substrates simplifies the manipulation of nanomaterial in a continuous process and reduces the loss of sorbent during column operations. The selective sorption of Cu^2+^ and Ni^2+^ is explained due to the soft–soft interaction between the metal ions and the nitrogen groups present in the composite. A surface complexation inside the inner well explained the kinetics of the interaction between the metal ions and CNF@C_3_N_4_. The method can be used for concentrating small amounts of metal ions in water samples, with a limit of detection and a limit of quantification of 0.06 μg/L and 0.20 μg/L, respectively [54].

Nanocellulose/MOF materials were reported for heavy metal adsorption. Composites of cellulose–MOFs (referred to as CelloMOF) have been extensively documented for their ability to adsorb metal ions [91]. A study described the use of a foam composed of MIL-100(Fe) and BC for the adsorption of As^3+^ ions. The foam had a maximum adsorption capacity of 4.81 mg/g [92]. Zinc oxide nanorods put onto a cotton fabric exhibited improved adsorption effectiveness due to the inclusion of MOF materials, specifically ZIF-8 [93]. The materials ZIF-8@ZnO@cotton and ZnO@cotton exhibited absorption efficiencies of 70% and 38%, respectively [93]. The significant adsorption effectiveness is mostly attributed to the extensive surface area of MOF materials. The adsorption of Pb^2+^ ions was achieved using magnetic cellulose nanocrystals (MCNCs) and a Zn-based MOF [94]. The MCNC@Zn-MOF and MCNC exhibited adsorption capabilities of 558.6 and 92.2 mg/g, respectively [94]. Cellulose additionally enhances the adsorption capabilities of MOF compounds. A combination of ZIF-8 and BC exhibited a 1.2-fold increase in adsorption capacity for Pb^2+^ ions compared with pure ZIF-8 [95]. Additionally, cellulose provides a material based on MOF with a structure that is both lightweight and permeable [96,97,98]. Cellulose-MOF materials can be fabricated as aerogels [99,100,101], filter paper [102], substrate [103], membrane [104], and foams [105]. Cellulose/MOFs are active materials for water treatment [106].

Metal ion adsorption using cellulose–metal oxide nanocomposites has been published. Nanocellulose extracted from rice husk and sugarcane was modified with iron oxide nanocomposites (NIONs) for the adsorption of As^3+^ ions [107]. Fe_2_O_3_/CNs were synthesized using the solvothermal method to achieve maximum adsorption of As^3+^ and As^6+^ ions [108]. At pH 7 and 3, the adsorption capacities were 13.866 mg/g and 15.712 mg/g, respectively [108]. Magnetic attapulgite/chitosan/BCNs were used for the adsorption of Pb^2+^, Cu^2+^, and Cr^6+^ [109]. These materials can be easily recovered using an external magnetic field and can be recycled multiple times with less than an 8% decrease in performance after five cycles of adsorption-desorption [109]. Magnetic nanoparticles offer advantages such as simple separation using an external magnetic field [110]. For adsorbing Ni^2+^ and Co^2+^ ions, a hybrid membrane made of cellulose membrane and Bi_4_O_5_Br_2_/BiOBr nanosheets was described [111]. Mixed materials of BiOBr (JCPDS-01-085-0862) and Bi_4_O_5_Br_2_ (JCPDS-37-0699) are present in the produced membrane [111]. The adsorption capabilities of Co^2+^ and Ni^2+^ were 28.7 and 29.7 mg/g, respectively, and with CM/Bi_4_O_5_Br_2_/BiOBr, the values were 37.3 and 30.2 mg/g, respectively [111]. A combination of cellulose and ZrO_2_ exhibited preferential adsorption toward Ni^2+^ [112]. The challenge of using metal oxide is the decomposition of the adsorbent under an acidic environment that is present mainly in water contaminated with heavy metals.

**Table 2:** Examples of pollutant removal using nanocellulose-based materials.

Materials	Prepared Methods	Source	Pollutants	Techniques	Conditions	Capacity (mg/g)	Efficiency (%)	Ref.
NOCNF	Acid hydrolysis	Grass from Australia, named 104 spinifex	Cd^2+^	Adsorption	Cd^2+^ concentration of 250 ppm, 25 °C	2550	84	[38]
CNF@C_3_N_4_	Salt melt approach	Commercial	Ni^2+^ and Cu^2+^	Separation	Adsorbent, 500 mg; pH of 6.0, 100 mL of the solution containing metal ions	340 385	99.5 99.2	[54]
MIP-NC	Acid hydrolysis; reversed-phase microemulsion method	Cotton wool	Pb^2+^ Hg^2+^	Adsorption	pH 6,	27.55 161.31		[88]
CE-SPES	Base and acid extraction; stirring	Rice waste	Zn^2+^	Adsorption	Adsorbent, 0.02 g, pH = 5; 30 min, T = 30 °C for SPES; and Zn^2+^ concentration = 2 ppm	5.96	80.4–96.08	[89]
Cellulose methacrylate hydrogels	Extraction self-crosslinking thiol-ene click chemistry	Wood	Dyes: methyl blue and methylene blue	Adsorption	Hydrogel, 20 mg; dye, 1000 mg·L^−1^; 25 °C	934.63 mg·g^−1^ 706.64 mg·g^−1^	98.15–90.90	[113]
Ag/lignin/cellulose	Freezing-thawing process	Commercial	dyes, pollutants, and antibiotics	Catalytic reduction	2 mM NaBH_4_ (75 mg) or 100 mg of studied aerogels, 25 mg/L MB solution, 30 min		99.8–99.9	[114]
MCNC	Hydrothermal	Commercial	DOX	Adsorption	0.1 g of samples, 100 mL of DOX solution (20 mg/L) at pH 7, 3 h at 300 rpm shaking	61.2 mg/m^2^	70	[115]
Sr/Alg/CMC/GO/TiO_2_	Hummers method freeze drying processes; cross-linking	Commercial	Dye: Congo red	Photocatalysis	1.2 g/L of photocatalyst, 30 mL of aqueous solution of CR dye (30 mg/L), for 2 min and stirred for 30 min under dark conditions, 900 W/m^2^	98%	240	[116]
CNC	Cotton pulp	Acid hydrolysis	*L* = 90 ± 10 nm, diameter, *D* = 8 ± 1 nm and zeta potential of 51.5 ± 0.8 mV	Bacteria	*Pseudomanas aeruginosa*	Flocculation	*P. aeruginosa* suspension, 1.0 × 10^8^ cells/mL in 10 mM NaCl solution, bacteria to CNC ratios of 1:100,000, 24 h	[117]
Imidazolyl CNCs	Cotton wool	Acid hydrolysis	DS, 0.06; pH adjusted to 10.8 using sodium carbonate (ζ = −16.1 ± 1.3 mV). pH = 5.7 and ζ = 9.9 ± 0.8 mV	microalgae	Chlorella Vulgaris	Flocculation	200 mg L^−1^ dose, 2–3% CO_2_, biomass concentration of 0.35 g L^−1^	[118]

### 6.2. Removal Anionic Species

Water pollution caused by anionic species, including fluoride (F^−^), sulfate (SO_4_^2−^), nitrate (NO_3_^−^), nitrite (NO_2_^−^), and phosphate (PO_4_^3−^), has negative impacts on human health. Anionic species, such as F^−^, pose a significant risk due to their ability to penetrate human skin and dissolve bone and teeth. They have the capability to transform hydroxyapatite (Ca_10_(PO_4_)_6_(OH)_2_) into fluorapatite (Ca_5_(PO_4_)_3_F). The rules set by the World Health Organization (WHO) specify that the maximum permissible level of fluoride ions in daily drinking water should not exceed 1.5 parts per million (ppm). Consuming water with high levels of sulfate ions (>600 ppm) can have a laxative effect. Anionic contaminants, such as sulfate, nitrate, nitrite, and phosphate ions, are highly soluble in water, making their removal a challenging process.

Anionic contaminants in water can be eliminated using various techniques such as precipitation and adsorption. The utilization of anionic adsorbents, like cellulose, leads to ineffective adsorption of anionic species. Therefore, cationic cellulose is extensively utilized for the adsorption of anionic contaminants. It can efficiently absorb anions such as nitrate and fluoride. A study found that the ability of quaternized cellulose nanofibrils to adsorb anionic dyes was enhanced when the amount of trimethylammonium chloride (TMAC) on the cellulose nanofibrils increased [119]. Positively charged quaternary ammonium groups (QCNF) were incorporated into cellulose nanofibers derived from waste pulp remnants [120]. The synthesis procedure consisted of the conversion of the pulp into ether by employing glycidyl trimethylammonium chloride, followed by the process of mechanical method. The substance that was created had a cationic charge concentration of 1.2 millimoles per gram. The QCNF material exhibited preferential adsorption for multivalent ions such as PO_4_^3−^ and SO_4_^2−^, as opposed to monovalent ions like F^−^ and NO_3_^−^ [120].

Cerium oxide (CeO_2_) nanoparticles were synthesized within a biomass-recyclable cellulose membrane (BCCM) to adsorb fluoride from industrial effluent [121]. The BCCM exhibited an adsorption capacity of 48.0 mg/g for fluoride ions. The membrane had superior adsorption capability in comparison to cellulose/hydroxyapatite (Hap) [122]. GO/CNFs/ZrO_2_ refers to self-assembled peptide nanofibers (PNFs) that have been manufactured using a biomineralization approach. These nanofibers, known as GO/PNF/CNF-ZrO_2_, are specifically designed for the adsorption of F^−^ ions [123]. The membrane exhibited a selectivity of 95.39%, achieving maximum adsorption of 11.63 mg/g. The adsorption capacity of the biohybrid membrane increases proportionally with the amount of ZrO_2_ present [123]. The material demonstrates a high level of sustainability based on the Overall Sustainability Footprint (OSF) technique, which takes into account performance, cost, and environmental impact. It has an OSF value of 83%, which is higher than other materials [123].

A study reported the use of CMC/citric acid (CA) aerogel for the removal of anionic contaminants such as nitrate, nitrite, and phosphate [124]. The aerogels demonstrated a notable specific surface area along with substantial porosity, resulting in a low density. Their adsorption capability for nitrite (NO_2_^−^), nitrate (NO_3_^−^), and phosphate (PO_4_^3−^) was demonstrated to be 79.65, 73.04, and 98.18 ppm, respectively [110]. Cellulose-based adsorbents are effective materials for fluoride ion removal [125].

The kinetic properties of boron adsorption on microcrystalline cellulose, lignin, and polymeric resin were compared [126]. Borate anions chemically bond with the neighboring diols in the glucopyranoside units of cellulose to form stable boron chelate complexes. Unlike cellulose, lignin shows no chelate complexes due to the lower concentration of cis-vicinal diols. The rates of formation and the stability of these chelate complexes are highly influenced by nanoscale features, as well as reaction circumstances such as pH and the concentration of the sorbate and sorbent [126].

Because of their negative charge, cellulose adsorption for anionic species is low and should be improved. For instance, MCC showed a boron adsorption capacity of 1.3–3.0 mg/g, which is lower than the boron adsorption capacity of Amberlite IRA 743, a polystyrene-based resin, which is around 17.2 mg/g [126].

### 6.3. Desalination: Salts Rejection

**Figure 6:**
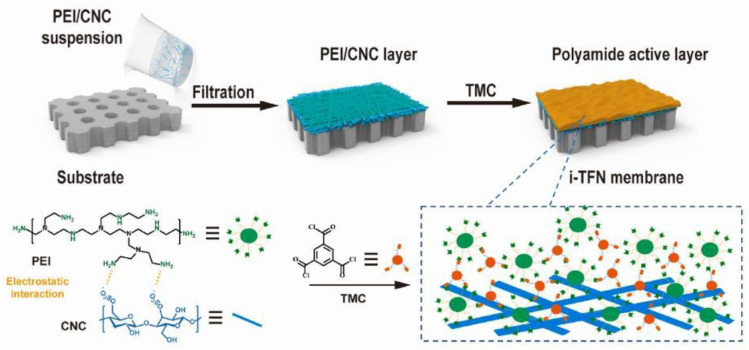
Preparation of PEI/CNC membrane and their salts rejection mechanism. Figure reprinted with permission from Ref. [127] Copyright© 2023 Elsevier B.V.

Cellulose-based materials were reported for desalination. They were used for thin film composite (TFC) of polyamide membranes. Cellulose-based membranes can be prepared using interfacial polymerization of reactive monomers (Figure 6) [127]. The synthesis procedure involved a complexation of amine monomers with CNC, and their subsequent deposition on a substrate for the polymerization reaction changes the polymerization process and creates a nanocomposite membrane structure with interlayered-thin films (i-TFNs). This technique may enhance the efficiency of amine loading and distribution for the interfacial polymerization reaction. Additionally, CNCs with desirable functional groups can restrict the release and diffusion of the amine monomer. As a consequence, a crumpled membrane structure is formed while maintaining a continuous polymer network. The membrane was investigated for salt rejections of MgCl_2_, MgSO_4_, NaCl, LiCl, and Na_2_SO_4_. The salt rejections can be ordered in the sequence of MgCl_2_ > MgSO_4_ > NaCl > LiCl > Na_2_SO_4_. The authors interpreted the order due to the combined effects of Donnan exclusion and steric hindrance [127]. The improved membrane exhibited a water permeance that was three times greater than the pristine membrane while maintaining the same level of salt rejection performance [127]. Nevertheless, the balance between the capacity of TFC membranes to allow substances to pass through and their ability to selectively separate different substances hinders any more improvements in separation efficiency. Cellulose-based materials can be used to solve these challenges and enable high performance.

### 6.4. Removal Organic-Based Pollutants: Adsorption and Catalysis

Cellulose modification enabled high dye adsorption. The cellulose methacrylate organogels were prepared via self-crosslinking and thiol-ene click chemistry [128]. The cellulose hydrogels exhibit high adsorption capacity for cationic and anionic dyes. The adsorption process depends on the presence of carboxyl or amine groups that were grafted onto the hydrogels. It was described by a Langmuir isotherm and a pseudo-second-order kinetic model [113]. A crosslinking strategy was used to prepare a cellulose-based aerogel adsorbent (CPB) for both static and dynamic dye removal [129]. The process involved the fabrication of cellulose acetoacetate (CAA) and β-cyclodextrin acetoacetate (β-CDAA) through a transesterification process. The polymer chains were then crosslinked using polyethyleneimine (PEI) through the formation of dynamic enamine bonds. The three-dimensional (3D) network structures were obtained using freeze-drying. The prepared hydrogel was evaluated for methyl orange (MO) adsorption, offering an adsorption capacity of 1013.11 mg/g. It possesses a high positive charge on its surface, offering high selectivity toward anionic dyes from ternary mixed systems [129].

**Figure 7:**
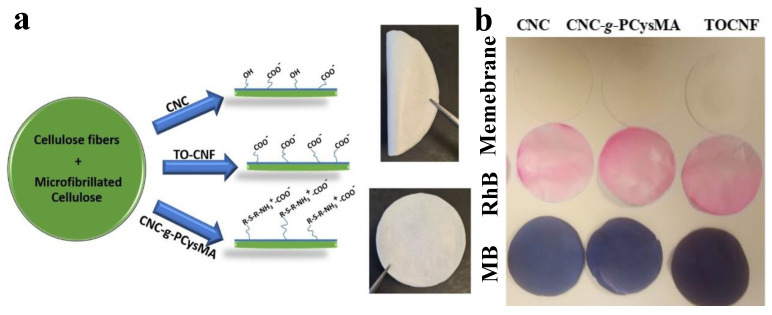
(**a**) synthesis of Cellulose membranes and (**b**) the adsorption of organic dyes, RhB, and MB. Figure reprinted from Ref. [49] This open-access article is licensed under a Creative Commons Attribution-NonCommercial 3.0 Unported License.

Electrospun fibers made of cellulose acetate (CA) and CNC, with varying weight percentages of CA: CNC [130], demonstrated a rejection efficacy of 20–56% for particles (size of 0.5 to 2.0 μm) and 80–99% for dyes [130]. Membranes composed of cellulose and other additives such as CNC, TOCNF, or CNC-g-PCysMA were developed to adsorb organic dyes, including rhodamine B (RhB) and methylene blue (MB, Figure 7b) [49]. The membranes were produced using a sheet forming called Rapid-Köthen (Figure 7a). Their flux ranged from 3417 to 11,742 L/h·m^2^·bar^−1^, demonstrating a high level of performance. MB can be removed using adsorption, with CNC, CNC-g-PCysMA, and TOCNF exhibiting adsorption efficiencies of 26%, 21%, and 35%, respectively (Figure 7b). In addition, they can catalyze the process of dye decolorization through hydrogenation with the aid of sodium borohydride (NaBH_4_). The membrane exhibits a decolorization efficacy of 100% towards RhB [49]. Nevertheless, this process can be reversed because the dye is not completely degraded (Figure 7). Cellulose underwent modification using several polymers, including polypiperazine [131] and hydrolyzed polyacrylamide (HPAM), which effectively facilitated the removal of MB [132]. CaCO_3_ nanoparticles, known as pore-forming agents or porogens, can be incorporated into the cellulose/MFC composites during their development. These nanoparticles can then be eliminated under acidic circumstances following the production of the MCMFC composite, allowing for precise control over the porosity [133].

Nanocellulose is suitable to support for catalysts [134]. A composite of AgNPs/lignin/cellulose was prepared via the chemical reduction of silver ions (Ag^+^) [114]. The reduction process produced metallic silver nanoparticles with a size smaller than 40 nm [114]. The Ag/lignin nanoparticles were physically interconnected inside the cellulose hydrogel and then subjected to freeze-drying to provide the Ag/lignin nanoparticle-loaded cellulose aerogel. The hydrogels were used for water treatment by removing pollutants such as dyes, drugs, and antibiotics. The degradation was achieved using in situ-generated hydrogen via the hydrolysis of NaBH_4_ and natural sunshine radiation. The catalytic process enabled the removal of 99.8%, 99.9%, 99.9%, 99.5%, and 99.8% for methylene blue, methyl orange, rhodamine B, 4-nitrophenol, and doxycycline hyclate, respectively [114]. The intrinsic cytotoxicity of nanoparticles such as silver nanoparticles should be considered when applying them for water treatment.

Metallic oxide nanoparticles modified cellulose-based materials were reported for the removal of organic pollutants, including pharmaceuticals, drugs, and dyes. A magnetic cellulose nanocrystal (MCNC) was synthesized from microcrystalline cellulose (MCC) using a one-pot hydrothermal method [115]. The synthesis included the addition of chemical reagents such as the presence of FeCl_3_, FeCl_2_, urea, and hydrochloric acid in the presence of cellulose materials. MCNC was modified via post-treatment procedure using chloroacetic acid (CAA), chlorosulfonic acid (CSA), or iodobenzene (IB). MCNC enhanced the adsorption efficiency of doxycycline hyclate (DOX) [115].

A 3D cross-linked porous structure for an amino-functionalized BC/Ti_3_C_2_T_x_ MXene (ABC/MX) composite was reported for metal and dye removal [135]. The successful insertion of amino groups not only increases the active sites accessible for adsorption but also improves the interfacial contacts between BC and Ti_3_C_2_T_x_. The findings demonstrate the remarkable removal effectiveness of the ABC/MX composite, with maximal adsorption capacities of 200.7 and 1103.7 mg/g for Cr(VI) and Congo red (CR), respectively. The composite offered several active sites that enabled chelation, hydrogen bonding, reduction reactions, and electrostatic interactions with water pollutants [135]. The incorporation of MXene and cellulose addresses the inadequate mechanical characteristics of pure MXene and enhances the performance of composite materials. MXene/cellulose composites offered high mechanical, stability, and electrical properties. A review was published for MXene/cellulose composites in Ref. [136] It also offers a comprehensive description of their various applications, such as electromagnetic shielding, intelligent electronics, energy storage, and water treatment [136].

Metallic oxide-modified cellulose materials are effective catalysts for organic pollutant removal. A flexible and recyclable cellulose-based membrane was reported for removing organic pollutants such as dyes [137]. It was synthesized using a combination of silica-coated BC scaffolds and titania [137]. The BC scaffolds have both macroscopic and nanometric internal pores, making them suitable as functional supports. By incorporating silica and titania, the resulting hybrid organic-inorganic aerogel membranes are self-standing, porous, and capable of effectively removing organic pollutants through photo-assisted processes [137]. The hybrid aerogels were synthesized by sequentially depositing a SiO_2_ layer over BC, then coating the resulting BC@SiO_2_ membranes with a porous titania aerogel overlayer. This process involved epoxide-driven gelation, hydrothermal crystallization, and subsequent supercritical drying, resulting in a high surface area. The BC@SiO_2_-TiO_2_ hybrid aerogel exhibited significantly enhanced photocatalytic activity, achieving up to 12 times quicker elimination of methylene blue dye from aqueous solution compared with bare BC/TiO_2_ aerogels [137]. The degradation of Congo red (CR) dye using direct sunshine was also reported using a nanocomposite material consisting of Sr^2+^-crosslinked alginate/carboxymethyl cellulose (Alg/CMC) with included TiO_2_ nanoparticles and graphene oxide (GO) [116]. Sr/Alg/CMC/GO/TiO_2_ offered a higher degradation efficiency of 98% compared with the Sr/Alg/CMC/TiO_2_, Sr/Alg/CMC, and bare TiO_2_ photocatalysts, with efficiencies of 70%, 60%, and 62%, respectively [116].

Metal chalcogenides were impregnated into cellulose materials. A straightforward impregnation–precipitation method was used to create the CdS/mixed cellulose composite membrane (CdS/MCM) [138]. MCM prevents the aggregation of CdS nanoparticles without obstructing the membrane channels. It also enhanced the catalytic performance of CdS, offering higher photocatalytic properties than CdS powder. CdS/MCM offered 96% degradation efficiency of potassium butyl xanthate, potassium amyl xanthate, and sodium ethyl xanthate [138]. Copper sulfide (CuS)/cellulose composite was reported for solar steam evaporator [139]. CuS was located at the top surface of the composite hydrogel and developed a heating zone under simulated solar light. The composite offered an evaporation rate and solar-to-vapor efficiency of 2.2 kg/m^2^·h and 87%, respectively, under one sun irradiation. It showed high stability under radiation for 48 h with an evaporation rate of 2.1 kg/m^2^·h. It demonstrated a 95% and 92% efficient photocatalytic degradation of methylene blue and rhodamine B, respectively [139].

**Figure 8:**
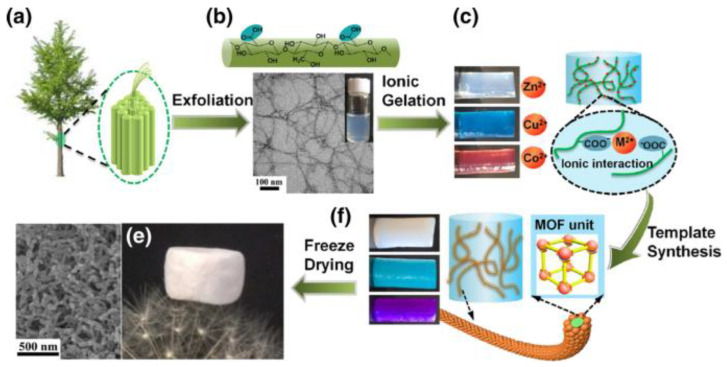
The synthesis of MOF aerogels with the template of CNFs. Figure reprinted with permission from Ref. [140] Copyright© 2018 American Chemical Society.

Cellulose enables the processing of MOF powders into micro/mesoporous crystalline structures (Figure 8) [140]. Fibrous MOF aerogels were created by synthesizing MOF crystals on TEMPO-cellulose nanofibrils via the three-dimensional coordination of metal nodes and organic linkers (Figure 8). The synthesis procedure offered aerogel with macroscopic shapeability and extrinsic hierarchical porosity (99%) with low density (0.1 g/cm^3^, Figure 8). Cellulose nanofibrils provided both external porosities and mechanical flexibility to the resulting MOF aerogels. Additionally, they altered the equilibrium between nucleation and growth, leading to the synthesis of smaller MOF crystals and reducing the likelihood of aggregation. The aerogel can be used for dye adsorption [140].

**Figure 9:**
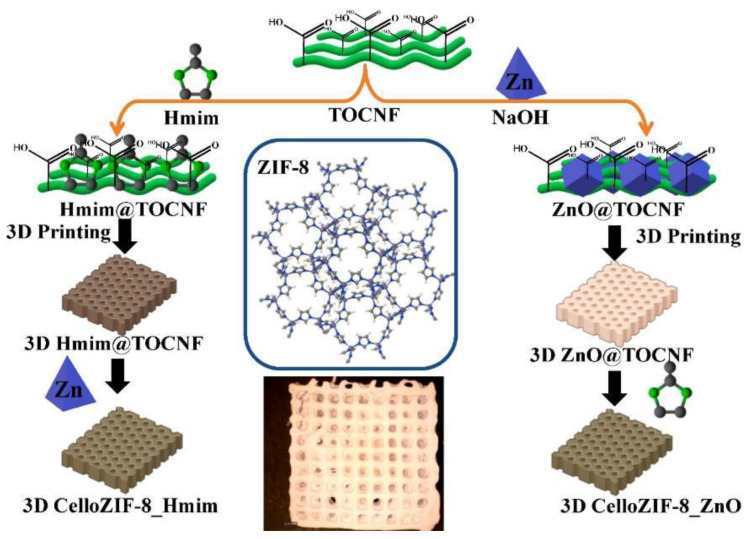
Three-dimensional printing of CelloZIF-8. Figure reprinted with permission from Ref. [55], open access.

Nanocellulose using TOCNF enabled the processing of MOF material such as ZIF-8 via three-dimensional (3D) printing (Figure 9) [55]. The procedure is a free-binder method for 3D printing of CelloZIF-8 materials. It offered a high loading of 67.5 wt.% of ZIF-8. It can be used for 3D printing of hierarchical porous. The printing structures were applied as adsorbents for dye and metal ions. They were also applied as catalysts for the degradation of organic dyes, offering high adsorption capacity (8–328 mg/g) and degradation efficiencies (>99%) within 10 min [55]. A membrane of cellulose acetate (CA)/ZIF-8 was also reported for nanofiltration of bovine serum albumin and dye removal [141]. Data analysis showed flux recovery ratio values of 85% and 90% for the bare CA and CA/ZIF-8 membranes, respectively. The dye removal efficiency was 97.7% for Reactive Black 5 dye [141]. A bead adsorbent was reported using regenerated cellulose hydrogel and activated carbon based on ZIF-8 (AC-ZIF-8) was combined to create a bead-type adsorbent [142]. Large surface area AC-ZIF-8 particles were produced by carbonization and chemical activation using KOH. Using cellulose dissolution/regeneration, the AC-ZIF-8 powders were effectively immobilized in hydrophilic cellulose hydrogel beads. AC-ZIF-8/cellulose hydrogel (AC-ZIF-8/CH) composite beads have an adsorption capacity of 565.13 mg/g for Rhodamine B [142]. Furthermore, the AC-ZIF-8/CH beads worked well with a variety of dyes and across a wide pH and temperature range [142].

Quaternizated porous cellulose beads were reported via modification with glycidyltrimethylammonium chloride [143]. The beads were investigated for removing humic acid (HA) from water [143]. The adsorption process of humic acid followed the pseudo-second-order kinetic model and showed a stronger correlation with the Langmuir isotherm model. This indicates that the adsorption of HA occurs through the electrostatic interaction between a quaternary ammonium group and a dissociated carboxy group of an HA molecule [143].

Water pollutants can be removed using flocculants that induce the aggregation of suspended particles, resulting in the formation of bigger flocs. Subsequently, these clusters are more readily eliminated from the water via the processes of sedimentation or filtering. Typically, natural polymer-based flocculants may be divided into two types: (1) semi-natural, such as polyacrylamide-grafted hydroxypropyl methyl cellulose, and (2) dicarboxylic acid nanocellulose. Bio-based flocculants such as cellulose have several advantages, such as biodegradability, a large specific surface area, and diameters in the nanoscale domain.

### 6.5. Bacteria Removal from Wastewater

Numerous research has concentrated on altering the physical and chemical structure to enhance the qualities of nanocellulose. CNCs were reported to flocculate Gram-negative bacteria, namely *Pseudomonas aeruginosa* PAO1, with an average length of 1.2 μm and a width of 0.2 μm and zeta potential of −29.84 ± 0.6 mV [117]. A rod-shaped CNC was investigated with a length (L) of 90 ± 10 nm, diameter (D) of 8 ± 1 nm, and zeta potential of 51.5 ± 0.8 mV. The phenomenon of bacterial flocculation occurred when the concentration of CNC was below 0.1%, which can be attributed to the depletion effect. The rod-shaped nanoparticles of CNCs were highly efficient in causing the aggregation of bacteria of colloidal size through depletion flocculation. Additionally, the authors observed that the separation of bacteria into distinct phases can occur even at extremely low concentrations of rod-like CNC particles [117].

The performance of cellulose to remove microorganisms was improved using several methods, including (1) cellulose modifications and (2) the improvement of experimental conditions. The incorporation of cationic pyridinium [144] and imidazole [118] functional groups via CNC grafting improved the performance compared with conventional polymer flocculants in terms of their ability to efficiently flocculate negatively charged microalgal cells. The use of gases such as CO_2_ can be used to control the flocculation of microalgae using CNCs [118].

An imidazole-grafting CNC, imidazolyl cellulose nanocrystals, was reported with a degree of substitution (DS, number of hydroxyls modified per anhydro-glucose-unit) of 0.06 via a one-pot procedure [118]. The prepared nanocrystals exhibited a surface charge that was responsive to changes in pH. Specifically, the surface charge was positive when the pH was below 6 and negative when the pH was above 7. The flocculation potential of the material was evaluated for *Chlorella vulgaris* utilizing CO_2_ as a flocculation inducer. A flocculation effectiveness of up to 90% was attained with a dosage of 200 mg/L. The materials can be used for developing reversible flocculation systems [118].

### 6.6. Nanocellulose for Oil Removal

Cellulose-based membranes are widely used for oil removal [145]. A membrane composite of CNF was reported containing pulp, cerium oxide (CeO_2_) nanoparticles, and stearic acid (STA), denoted as Pulp/CNF-CeO_2_-STA [146]. The synthesis procedure offered straightforward steps, including the coating method. The prepared membrane offered high super-wetting ability (water contact angle of 166 ± 2°) for oil-water separation application. Pulp/CNF-CeO_2_-STA membrane showed a high separation efficiency of 88%. It can be used for a high throughput of 73 L/m^2^·h. It can also be applied for photocatalytic degradation, offering a dye removal efficiency of 94% in water. The membrane exhibited good antimicrobial activity and offered the potential to serve as anti-bioadhesive material against *Chlorella* [146].

The combination of polyethersulfone (PES), cellulose acetate (CA), and 4A zeolite was used to create asymmetric membranes via the phase inversion technique [147]. These membranes were then tested for their effectiveness in treating oily wastewater. Kerosene was selected as a representative example of a polluting oil. The membranes were evaluated based on their water permeation, oil permeation flux, oil rejection, flux recovery ratio, and relative flux decrease. The ZPC membranes exhibited superior performance compared with the other membranes that were manufactured. The addition of 4A zeolite nanoparticles at a concentration of 0.5 wt.% to the PES/CA-blended membrane resulted in a substantial improvement in microfiltration performance and a reduction in the contact angle of the P membrane in a range of 70–29.8°. An investigation was conducted to examine the impact of operational factors, including transmembrane pressure (ranging from 1 to 4 bar), feed temperature (ranging from 25 to 50 °C), and concentration of oil feed solution (250 to 1000 mg/L), on the permeation flux and oil rejection of a PES/CA-blended membrane that contains 0.5 wt.% 4A zeolite (0.5 wt.%ZPC). The membrane with a composition of 0.5%ZPC demonstrated enhanced porosity of 87.7%. It achieved the greatest pure water flux value, oil permeation flux, maximum oil rejection, and flux recovery ratio of 91.1 L/m^2^·h, 75.55 L/m^2^·h, 98.8%, and 97.7%, respectively. The permeation flux provided by the 0.5%ZPC membrane was enhanced eight-fold compared with the P membrane. The membranes exhibited good re-usability for the long-lasting measurements [147].

## 7. Key Parameters Affecting Adsorption and Mechanism of Pollutant Removal

### 7.1. Functional Groups

Chemical modification of cellulose with suitable functional groups enabled selective adsorption of anionic and cationic dyes [128]. The presence of vicinal hydroxyl groups enabled selective adsorption of anionic species such as boron. The cis diols offered the formation of borates chelates. Xylan hemicellulose-based hydrogels were reported for the removal of metal ions contaminants, including Cd^2+^, Cu^2+^, and Pb^2+^, from an aqueous solution [148]. It was extracted from bleached kraft pulp of eucalyptus and then modified with functional sulfonic acid groups and sulfonate groups. The hydrogels exhibit a high ability to adsorb metal ions with a loading of 30–50% of xylan. Metal adsorption depends on the functional groups and the heavy metal ions [148].

### 7.2. Chemical Compositions

The presence of a silica layer between BC and the titania photocatalyst significantly affects the structure and composition of the hybrid aerogel membranes. This results in increased loading of TiO_2_ and the development of aerogel materials that are stable under photochemical conditions. These materials also have a larger surface area and pore volume, leading to higher photocatalytic activity [137].

### 7.3. Synthesis Procedure

The synthesis procedure affects the final products’ properties and their performance [149]. A study was conducted to investigate the effect of the preparation conditions for cellulose acetate membranes [150]. It reported the fabrication procedure using the phase inversion method. The authors investigated different conditions, such as the speeds of film casting and the thicknesses of the membranes. They changed the fabrication procedure via a coagulation bath and the addition of additives such as surfactant [150]. The synthesis conditions enabled membranes with a porosity ranging from 66 to 80% and an average pore size ranging from 0.017 to 0.060 µm. It affects the dye adsorption performance.

Treatment of membrane-based materials via pre- and post-synthetic procedures enabled high performance. Plasma treatment improved the performance of cellulose-based materials. Argon plasma treatment of cellulose nitrate membrane showed high permeate flux with a higher rejection rate [151]. The membranes have enlarged pores in size and area. The hydrophobic recovery for plasma-treated membranes was shown to be considerable after seven days of aging [151].

### 7.4. The Solution’s pH Values

The pH of the solution is a crucial factor in the adsorption process. The wastewater typically exhibits an acidic pH. Cellulose can serve as an efficient adsorbent, particularly when compared to other adsorption methods that exhibit instability in acidic environments. The adsorption of metals onto cellulose nanofibers typically occurs within a pH range of 3 to 7. To prevent the formation of metal ions as hydroxides or oxides, it is generally advisable to avoid pH values that are above 7, which are considered basic.

**Figure 10:**
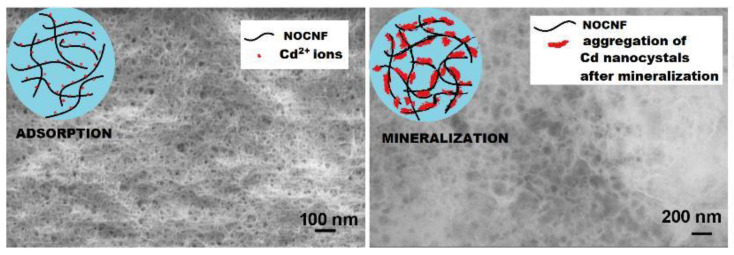
SEM images of carboxylated nanofibers (NOCNF) extracted from spinifex using a nitro-oxidation approach were efficient in removing cadmium (II) from the water via adsorption and mineralization. Figure reprinted with permission from Ref. [38] Copyright© 2018, American Chemical Society.

The chemical structure of cellulose offers an abundant amount of hydroxyl groups that may undergo chemical modifications to increase reactivity, binding capabilities, and mechanical durability. The mechanism depends on several parameters, including pollutant concentrations. The findings indicated that when Cd^2+^ concentrations were below 500 ppm, the primary remediation process included the interaction between carboxylate groups on the NOCNF surface and Cd^2+^ ions (Figure 10) [38]. Additionally, Cd^2+^ ions served as a cross-linking agent, causing the NOCNF solution to form a gel-like substance. At concentrations of Cd^2+^ over 1000 ppm, the primary mechanism for remediation was the formation of Cd(OH)_2_ nanocrystals via the process of mineralization. This was confirmed by the use of transmission electron microscopy (TEM) and wide-angle X-ray (WAXD) techniques [38].

The presence of heteroatom sites, such as nitrogen in the nanocellulose nanocomposite, enabled an interaction with the heavy metals. For instance, CNF/C_3_N_4_ offered nitrogen functional groups that enabled selective adsorption of Cu^2+^ and Ni^2+^ predominantly arising from the soft–soft interaction between metal ions and nitrogen functional groups [54].

**Figure 11:**
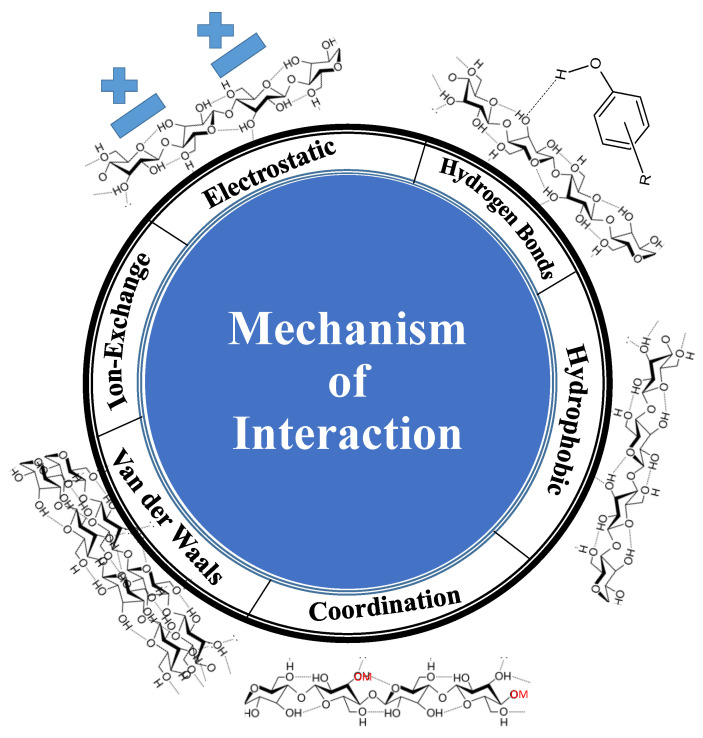
General mechanism for pollutant removal using cellulose-based materials.

There are several mechanisms for pollutant removal, as summarized in Figure 11. The mechanism depends on several parameters, such as adsorbent compositions, available functional groups in the materials, pollutant functional groups, and experimental conditions, as well as mechanisms such as electrostatics, hydrogen bonds, hydrophobic, van der Waals, ion exchanges, and coordination bonds. One or more than one mechanism can be present.

## 8. Advantages and Challenges

Nanocellulose can be produced via several sources, including biomass waste [152]. However, commercializing CNFs remains a significant problem. Other factors hinder the industrial production of CNFs. CNFs are primarily generated by the use of high-pressure homogenizers, high-energy ball mills (mechanical chemistry), microfluidizers, and ultra-low temperature crushing. Because CNFs are quite long and have large diameters, they possess a high specific surface area. This results in the creation of hydrogen bonds between the hydroxyl groups on the surface and the fibers, leading to the development of dense clumps that are difficult to separate. Relying only on mechanical power to cut and separate the fibers leads to substantial energy consumption. Moreover, the rapid and powerful heat generated during mechanical grinding directly affects the crystal structure of CNFs, resulting in the disruption of their network structure and modifying the gel behavior of the nanofibrils. Moreover, the cellulose fibrillation process necessitates being conducted in a liquid media. After the CNFs are dried, the fibers undergo irreversible hydrogen bonding, leading to a process called keratinization [153]. Consequently, CNF products are mostly present as aqueous dispersions, including small amounts of solid material. This necessitates a substantial allocation of storage capacity and results in elevated transportation expenses. To streamline the generation of CNFs, reduce the energy consumption associated with the procedure, and provide CNFs with a certain functional arrangement, a “two-step method” may be used for nanofibril manufacturing. This method entails an initial pre-treatment of cellulose, followed by a meticulous mechanical process to extract the nanofibrils by removing the microfibril structure of cellulose. There are three main steps involved in the preprocessing of cellulose: (i) Cellulase hydrolysis is employed to break down and separate the fibers, making it easier to mechanically separate them further; (ii) cellulose can be chemically altered by utilizing coupling agents (such as lauric acid, malic acid, etc.) to replace the hydroxyl groups on the cellulose surface or by interacting with hydroxyl groups to reduce the hydroxyl content on the cellulose surface. This alteration results in a decrease in the inter-cellulose and fiber content. The existence of hydrogen bonds and the inherent strength of the element aid in the disintegration of the fibers. Additionally, the hydroxyl groups on the surface of cellulose may be chemically modified by using an oxidant to convert them into aldehyde groups or carboxyl groups.

Nanocellulose can be modified with other materials, including inorganic nanoparticles [154]. It can also be proceeded into commercial and custom design products using several methods such as electrospinning [152] and 3D printing [57]. They can be fabricated into hydrogels, aerogels [155], and 3D structures or objects [57]. Thus, cellulose-based materials are good for filter fabrications [155].

## 9. Conclusions

This review explores the exciting potential of cellulose and its derivatives for removing wastewater pollutants. We discussed methods for the production and processing of nanocellulose. The focus then shifts to the power of cellulose-based materials like adsorbents, membranes, and aerogels in pollutant removal. Nanocellulose-based materials were reported as effective materials for removing pollutants like dyes, drugs, heavy metals, and bacteria cells. The removal process should include the science behind this removal process, including factors like kinetics and thermodynamics. Nanocellulose offers several advantages, such as high abundance, low environmental impact, and cost-effectiveness. Overall, this review paints a promising picture for the future of sustainable wastewater treatment using nanocellulose-based materials.

## Figures and Tables

**Table 1:** Summary of nanocellulose derivatives and their size.

Name	Diameter (nm)	Length (µm)	Aspect Ratio (L/D)
Cellulose Nanofibrils (CNFs)	2–20	100–1000	50–500
Cellulose Nanocrystals (CNCs)	5–20	100–500	20–50
Bacterial Nanocellulose (BNC)	10–100	1–100	10–100
Enzymatic Nanocellulose (ENC)	5–50	100–500	20–100
TEMPO-oxidized cellulose nanofibrils (TOCNFs)	2–10	100–1000	50–500

## Data Availability

All data are presented in the review.

## References

[1] Wu S., Shi W., Li K., Cai J., Chen L. (2022). Recent advances on sustainable bio-based materials for water treatment: Fabrication, modification and application. J. Environ. Chem. Eng..

[2] Zanaty M., Zaki A.H., El-Dek S.I., Abdelhamid H.N. (2024). Zeolitic imidazolate framework@hydrogen titanate nanotubes for efficient adsorption and catalytic oxidation of organic dyes and microplastics. J. Environ. Chem. Eng..

[3] Pérez J., Muñoz-Dorado J., de la Rubia T., Martínez J. (2002). Biodegradation and biological treatments of cellulose, hemicellulose and lignin: An overview. Int. Microbiol..

[4] Saito T., Nishiyama Y., Putaux J.-L., Vignon M., Isogai A. (2006). Homogeneous Suspensions of Individualized Microfibrils from TEMPO-Catalyzed Oxidation of Native Cellulose. Biomacromolecules.

[5] Pääkkö M., Ankerfors M., Kosonen H., Nykänen A., Ahola S., Österberg M., Ruokolainen J., Laine J., Larsson P.T., Ikkala O. (2007). Enzymatic Hydrolysis Combined with Mechanical Shearing and High-Pressure Homogenization for Nanoscale Cellulose Fibrils and Strong Gels. Biomacromolecules.

[6] Abdelhamid H.N., Mathew A.P. (2022). Cellulose-Based Nanomaterials Advance Biomedicine: A Review. Int. J. Mol. Sci..

[7] Etale A., Onyianta A.J., Turner S.R., Eichhorn S.J. (2023). Cellulose: A Review of Water Interactions, Applications in Composites, and Water Treatment. Chem. Rev..

[8] Goswami R., Singh S., Narasimhappa P., Ramamurthy P.C., Mishra A., Mishra P.K., Joshi H.C., Pant G., Singh J., Kumar G. (2024). Nanocellulose: A comprehensive review investigating its potential as an innovative material for water remediation. Int. J. Biol. Macromol..

[9] Mohammed N., Grishkewich N., Tam K.C. (2018). Cellulose nanomaterials: Promising sustainable nanomaterials for application in water/wastewater treatment processes. Environ. Sci. Nano.

[10] Jing L., Shi T., Chang Y., Meng X., He S., Xu H., Yang S., Liu J. (2024). Cellulose-based materials in environmental protection: A scientometric and visual analysis review. Sci. Total Environ..

[11] Das R., Lindström T., Sharma P.R., Chi K., Hsiao B.S. (2022). Nanocellulose for Sustainable Water Purification. Chem. Rev..

[12] Wang Z., Chen Z., Zheng Z., Liu H., Zhu L., Yang M., Chen Y. (2023). Nanocellulose-based membranes for highly efficient molecular separation. Chem. Eng. J..

[13] Payen A. (1838). Mémoire sur la composition du tissu propre des plantes et du ligneux. Comptes Rendus.

[14] Niemz P., Mai C., Schmitt U. (2023). Introduction to Wood Science. Springer Handbook of Wood Science and Technology.

[15] Sponsler O.L., Dore W.H. (1928). The structure of mercerized cellulose. I. The space lattice of mercerized ramie cellulose as developed from X-ray data 1. J. Am. Chem. Soc..

[16] Sharma R., Nath P.C., Mohanta Y.K., Bhunia B., Mishra B., Sharma M., Suri S., Bhaswant M., Nayak P.K., Sridhar K. (2024). Recent advances in cellulose-based sustainable materials for wastewater treatment: An overview. Int. J. Biol. Macromol..

[17] Lin N., Huang J., Dufresne A. (2012). Preparation, properties and applications of polysaccharide nanocrystals in advanced functional nanomaterials: A review. Nanoscale.

[18] Radoor S., Karayil J., Jayakumar A., Kandel D.R., Kim J.T., Siengchin S., Lee J. (2024). Recent advances in cellulose- and alginate-based hydrogels for water and wastewater treatment: A review. Carbohydr. Polym..

[19] Marchessault R.H., Morehead F.F., Walter N.M. (1959). Liquid Crystal Systems from Fibrillar Polysaccharides. Nature.

[20] Revol J.-F., Bradford H., Giasson J., Marchessault R.H., Gray D.G. (1992). Helicoidal self-ordering of cellulose microfibrils in aqueous suspension. Int. J. Biol. Macromol..

[21] Revol J.-F., Godbout L., Dong X.-M., Gray D.G., Chanzy H., Maret G. (1994). Chiral nematic suspensions of cellulose crystallites; phase separation and magnetic field orientation. Liq. Cryst..

[22] Kimura F., Kimura T., Tamura M., Hirai A., Ikuno M., Horii F. (2005). Magnetic Alignment of the Chiral Nematic Phase of a Cellulose Microfibril Suspension. Langmuir.

[23] Westerhoff P., Alvarez P., Li Q., Gardea-Torresdey J., Zimmerman J. (2016). Overcoming implementation barriers for nanotechnology in drinking water treatment. Environ. Sci. Nano.

[24] Tan K.B., Vakili M., Horri B.A., Poh P.E., Abdullah A.Z., Salamatinia B. (2015). Adsorption of dyes by nanomaterials: Recent developments and adsorption mechanisms. Sep. Purif. Technol..

[25] Santhosh C., Velmurugan V., Jacob G., Jeong S.K., Grace A.N., Bhatnagar A. (2016). Role of nanomaterials in water treatment applications: A review. Chem. Eng. J..

[26] Qu X., Alvarez P.J.J., Li Q. (2013). Applications of nanotechnology in water and wastewater treatment. Water Res..

[27] Wang D. (2019). A critical review of cellulose-based nanomaterials for water purification in industrial processes. Cellulose.

[28] Olivera S., Muralidhara H.B., Venkatesh K., Guna V.K., Gopalakrishna K., Yogesh Kumar K. (2016). Potential applications of cellulose and chitosan nanoparticles/composites in wastewater treatment: A review. Carbohydr. Polym..

[29] Thakur V.K., Voicu S.I. (2016). Recent advances in cellulose and chitosan based membranes for water purification: A concise review. Carbohydr. Polym..

[30] Yu J., Wang A.C., Zhang M., Lin Z. (2021). Water treatment via non-membrane inorganic nanoparticles/cellulose composites. Mater. Today.

[31] Qiu K., Netravali A.N. (2014). A Review of Fabrication and Applications of Bacterial Cellulose Based Nanocomposites. Polym. Rev..

[32] Oun A.A., Shankar S., Rhim J.-W. (2020). Multifunctional nanocellulose/metal and metal oxide nanoparticle hybrid nanomaterials. Crit. Rev. Food Sci. Nutr..

[33] Wei H., Rodriguez K., Renneckar S., Vikesland P.J. (2014). Environmental science and engineering applications of nanocellulose-based nanocomposites. Environ. Sci. Nano.

[34] Saud A., Saleem H., Khan A.W., Munira N., Khan M., Zaidi S.J. (2023). Date Palm Tree Leaf-Derived Cellulose Nanocrystal Incorporated Thin-Film Composite forward Osmosis Membranes for Produced Water Treatment. Membranes.

[35] Rånby B.G., Banderet A., Sillén L.G. (1949). Aqueous Colloidal Solutions of Cellulose Micelles. Acta Chem. Scand..

[36] Turbak A.K., Snyder F.W., Sandberg K.R. Microfibrillated Cellulose: Morphology and Accessibility (Conference)|OSTI.GOV. https://www.osti.gov/biblio/5039044-microfibrillated-cellulose-morphology-accessibility.

[37] Zimmermann T., Pöhler E., Geiger T. (2004). Cellulose Fibrils for Polymer Reinforcement. Adv. Eng. Mater..

[38] Sharma P.R., Chattopadhyay A., Sharma S.K., Geng L., Amiralian N., Martin D., Hsiao B.S. (2018). Nanocellulose from Spinifex as an Effective Adsorbent to Remove Cadmium(II) from Water. ACS Sustain. Chem. Eng..

[39] Onyianta A.J., Dorris M., Williams R.L. (2018). Aqueous morpholine pre-treatment in cellulose nanofibril (CNF) production: Comparison with carboxymethylation and TEMPO oxidisation pre-treatment methods. Cellulose.

[40] Rocha I., Ferraz N., Mihranyan A., Strømme M., Lindh J. (2018). Sulfonated nanocellulose beads as potential immunosorbents. Cellulose.

[41] dos Santos D.M., Leite I.S., Bukzem A.d.L., de Oliveira Santos R.P., Frollini E., Inada N.M., Campana-Filho S.P. (2018). Nanostructured electrospun nonwovens of poly(ε-caprolactone)/quaternized chitosan for potential biomedical applications. Carbohydr. Polym..

[42] Laitinen O., Suopajärvi T., Österberg M., Liimatainen H. (2017). Hydrophobic, Superabsorbing Aerogels from Choline Chloride-Based Deep Eutectic Solvent Pretreated and Silylated Cellulose Nanofibrils for Selective Oil Removal. ACS Appl. Mater. Interfaces.

[43] Ninomiya K., Abe M., Tsukegi T., Kuroda K., Tsuge Y., Ogino C., Taki K., Taima T., Saito J., Kimizu M. (2018). Lignocellulose nanofibers prepared by ionic liquid pretreatment and subsequent mechanical nanofibrillation of bagasse powder: Application to esterified bagasse/polypropylene composites. Carbohydr. Polym..

[44] Nakagaito A.N., Yano H. (2004). The effect of morphological changes from pulp fiber towards nano-scale fibrillated cellulose on the mechanical properties of high-strength plant fiber based composites. Appl. Phys. A Mater. Sci. Process..

[45] Dufresne A. (2013). Nanocellulose: A new ageless bionanomaterial. Mater. Today.

[46] Henriksson M., Henriksson G., Berglund L.A., Lindström T. (2007). An environmentally friendly method for enzyme-assisted preparation of microfibrillated cellulose (MFC) nanofibers. Eur. Polym. J..

[47] Berghem L.E.R., Pettersson L.G. (1973). The Mechanism of Enzymatic Cellulose Degradation. Eur. J. Biochem..

[48] Gatenholm P., Klemm D. (2010). Bacterial Nanocellulose as a Renewable Material for Biomedical Applications. MRS Bull..

[49] Georgouvelas D., Abdelhamid H.N., Li J., Edlund U., Mathew A.P. (2021). All-cellulose functional membranes for water treatment: Adsorption of metal ions and catalytic decolorization of dyes. Carbohydr. Polym..

[50] Aguilar-Sanchez A., Jalvo B., Mautner A., Rissanen V., Kontturi K.S., Abdelhamid H.N., Tammelin T., Mathew A.P. (2021). Charged ultrafiltration membranes based on TEMPO-oxidized cellulose nanofibrils/poly(vinyl alcohol) antifouling coating. RSC Adv..

[51] Bagheri A.R., Aramesh N., Lee H.K. (2022). Chitosan- and/or cellulose-based materials in analytical extraction processes: A review. TrAC Trends Anal. Chem..

[52] Georgouvelas D., Abdelhamid H.N., Edlund U., Mathew A.P. (2023). In situ modified nanocellulose/alginate hydrogel composite beads for purifying mining effluents. Nanoscale Adv..

[53] Soliman M., Sadek A.A., Abdelhamid H.N., Hussein K. (2021). Graphene oxide-cellulose nanocomposite accelerates skin wound healing. Res. Vet. Sci..

[54] Haseen U., Kapoor S., Khan R.A., Ahmad H., Koo B.H. (2024). In Situ Fabrication and Characterization of g-C3N4 onto Cellulose Nanofibers and Selective Separation of Heavy Metal Ions. ACS Omega.

[55] Nasser Abdelhamid H., Sultan S., Mathew A.P. (2023). Binder-free Three-dimensional (3D) printing of Cellulose-ZIF8 (CelloZIF-8) for water treatment and carbon dioxide (CO_2_) adsorption. Chem. Eng. J..

[56] Abdelhamid H.N. (2023). MOFTextile: Metal-Organic Frameworks Nanosheets Incorporated Cotton Textile for Selective Vapochromic Sensing and Capture of Pyridine. Appl. Organomet. Chem..

[57] Abdelhamid H.N., Sultan S., Mathew A.P. (2023). 3D printing of cellulose/leaf-like zeolitic imidazolate frameworks (CelloZIF-L) for adsorption of carbon dioxide (CO_2_) and heavy metal ions. Dalt. Trans..

[58] Nasser Abdelhamid H., Georgouvelas D., Edlund U., Mathew A.P. (2022). CelloZIFPaper: Cellulose-ZIF Hybrid Paper for Heavy Metal Removal and Electrochemical Sensing. Chem. Eng. J..

[59] Nasser Abdelhamid H., Mathew A.P. (2021). Cellulose-zeolitic imidazolate frameworks (CelloZIFs) for multifunctional environmental remediation: Adsorption and catalytic degradation. Chem. Eng. J..

[60] Zhu C., Liu P., Mathew A.P. (2017). Self-Assembled TEMPO Cellulose Nanofibers: Graphene Oxide-Based Biohybrids for Water Purification. ACS Appl. Mater. Interfaces.

[61] Ray S.S., Iroegbu A.O.C. (2021). Nanocellulosics: Benign, Sustainable, and Ubiquitous Biomaterials for Water Remediation. ACS Omega.

[62] Liu H., Liu Z., Hui L., Liu H., Liu P., Zhang F., An X., Wen Y., Wu S. (2019). Cationic cellulose nanofibers as sustainable flocculant and retention aid for reconstituted tobacco sheet with high performance. Carbohydr. Polym..

[63] Nkalane A., Oyewo O.A., Leswifi T., Onyango M.S. (2019). Application of coagulant obtained through charge reversal of sawdust-derived cellulose nanocrystals in the enhancement of water turbidity removal. Mater. Res. Express.

[64] Oyewo O.A., Mutesse B., Leswifi T.Y., Onyango M.S. (2019). Highly efficient removal of nickel and cadmium from water using sawdust-derived cellulose nanocrystals. J. Environ. Chem. Eng..

[65] Dogan H., Hilmioglu N.D. (2010). Zeolite-filled regenerated cellulose membranes for pervaporative dehydration of glycerol. Vacuum.

[66] Ali I., Gupta V.K. (2006). Advances in water treatment by adsorption technology. Nat. Protoc..

[67] Singh N.B., Nagpal G., Agrawal S. (2018). Rachna Water purification by using Adsorbents: A Review. Environ. Technol. Innov..

[68] Faiz Norrrahim M.N., Mohd Kasim N.A., Knight V.F., Mohamad Misenan M.S., Janudin N., Ahmad Shah N.A., Kasim N., Wan Yusoff W.Y., Mohd Noor S.A., Jamal S.H. (2021). Nanocellulose: A bioadsorbent for chemical contaminant remediation. RSC Adv..

[69] Doyo A.N., Kumar R., Barakat M.A. (2023). Recent advances in cellulose, chitosan, and alginate based biopolymeric composites for adsorption of heavy metals from wastewater. J. Taiwan. Inst. Chem. Eng..

[70] Motloung M.T., Magagula S.I., Kaleni A., Sikhosana T.S., Lebelo K., Mochane M.J. (2023). Recent Advances on Chemically Functionalized Cellulose-Based Materials for Arsenic Removal in Wastewater: A Review. Water.

[71] Rana A.K., Gupta V.K., Hart P., Thakur V.K. (2024). Cellulose-alginate hydrogels and their nanocomposites for water remediation and biomedical applications. Environ. Res..

[72] Sharma P.R., Sharma S.K., Borges W., Chen H., Hsiao B.S. (2020). Remediation of UO22+ from Water by Nitro-Oxidized Carboxycellulose Nanofibers: Performance and Mechanism. Contaminants in Our Water: Identification and Remediation Methods.

[73] Sharma S.K., Sharma P.R., Chen H., Johnson K., Zhan C., Wang R., Hsiao B. (2020). Cellulose-Supported Nanosized Zinc Oxide: Highly Efficient Bionanomaterial for Removal of Arsenic from Water. Contaminants in Our Water: Identification and Remediation Methods.

[74] Chen H., Sharma S.K., Sharma P.R., Yeh H., Johnson K., Hsiao B.S. (2019). Arsenic(III) Removal by Nanostructured Dialdehyde Cellulose–Cysteine Microscale and Nanoscale Fibers. ACS Omega.

[75] Qin F., Fang Z., Zhou J., Sun C., Chen K., Ding Z., Li G., Qiu X. (2019). Efficient Removal of Cu^2+^ in Water by Carboxymethylated Cellulose Nanofibrils: Performance and Mechanism. Biomacromolecules.

[76] Movaghgharnezhad S., Mirabi A., Toosi M.R., Rad A.S. (2020). Synthesis of cellulose nanofibers functionalized by dithiooxamide for preconcentration and determination of trace amounts of Cd(II) ions in water samples. Cellulose.

[77] Awang N.A., Wan Salleh W.N., Ismail A.F., Yusof N., Aziz F., Jaafar J. (2019). Adsorption Behavior of Chromium(VI) onto Regenerated Cellulose Membrane. Ind. Eng. Chem. Res..

[78] Sharma P.R., Sharma S.K., Nolan M., Li W., Kundal L., Hsiao B.S. (2021). Sequential Oxidation on Wood and Its Application in Pb^2+^ Removal from Contaminated Water. Polysaccharides.

[79] Hernández-Francisco E., Bonilla-Cruz J., Márquez-Lamas U., Suárez-Jacobo Á., Longoria-Rodríguez F., Rivera-Haro J., Russell P., Ali Z., Yin C.-Y., Lara-Ceniceros T.E. (2020). Entangled cellulose nanofibrils/nanosheets derived from native mexican agave for lead(II) ion removal. Cellulose.

[80] Chen H., Sharma S.K., Sharma P.R., Chi K., Fung E., Aubrecht K., Keroletswe N., Chigome S., Hsiao B.S. (2021). Nitro-oxidized carboxycellulose nanofibers from moringa plant: Effective bioadsorbent for mercury removal. Cellulose.

[81] Zhu C., Monti S., Mathew A.P. (2020). Evaluation of nanocellulose interaction with water pollutants using nanocellulose colloidal probes and molecular dynamic simulations. Carbohydr. Polym..

[82] Kardam A., Raj K.R., Srivastava S., Srivastava M.M. (2014). Nanocellulose fibers for biosorption of cadmium, nickel, and lead ions from aqueous solution. Clean Technol. Environ. Policy.

[83] Chen B., Zheng Q., Zhu J., Li J., Cai Z., Chen L., Gong S. (2016). Mechanically strong fully biobased anisotropic cellulose aerogels. RSC Adv..

[84] Karim Z., Monti S. (2021). Microscopic Hybrid Membranes Made of Cellulose-Based Materials Tuned for Removing Metal Ions from Industrial Effluents. ACS Appl. Polym. Mater..

[85] Liu P., Sehaqui H., Tingaut P., Wichser A., Oksman K., Mathew A.P. (2014). Cellulose and chitin nanomaterials for capturing silver ions (Ag^+^) from water via surface adsorption. Cellulose.

[86] Yu X., Tong S., Ge M., Wu L., Zuo J., Cao C., Song W. (2013). Adsorption of heavy metal ions from aqueous solution by carboxylated cellulose nanocrystals. J. Environ. Sci..

[87] Rocky M.M.H., Rahman I.M.M., Biswas F.B., Rahman S., Endo M., Wong K.H., Mashio A.S., Hasegawa H. (2023). Cellulose-based materials for scavenging toxic and precious metals from water and wastewater: A review. Chem. Eng. J..

[88] Liu S. (2023). Preparation of nanocellulose grafted molecularly imprinted polymer for selective adsorption Pb(II) and Hg(II). Chemosphere.

[89] Mustafa F.H.A., Attia H.A.E.-A., Yahya R., Elshaarawy R.F.M., Hassan N. (2022). Cellulose microfibrils-embedded sulfonated polyethersulfone for efficient Zn^2+^ ions removal from aqueous effluents. Chem. Eng. Res. Des..

[90] Patel K., Sutar A.K., Maharana T. (2022). Synthesis of carboxylic graphene oxide-carboxymethyl chitosan composite and its applications toward the remediation of U^6+^, Pb^2+^, Cr^6+^, and Cd^2+^ ions from aqueous solutions. J. Chin. Chem. Soc..

[91] Xiao Z., Zhou J., Fan L., Li Y., He Y., Wang Y., Li L. (2021). Controllable Preparation of Cu-MOF-Coated Carboxyl Filter Paper for Simultaneous Removal of Organic Dye and Metal Ions. Ind. Eng. Chem. Res..

[92] Ashour R.M., Abdel-Magied A.F., Wu Q., Olsson R.T., Forsberg K. (2020). Green Synthesis of Metal-Organic Framework Bacterial Cellulose Nanocomposites for Separation Applications. Polymers.

[93] Schelling M., Kim M., Otal E., Aguirre M., Hinestroza J.P. (2020). Synthesis of a zinc–imidazole metal–organic framework (ZIF-8) using ZnO rods grown on cotton fabrics as precursors: Arsenate absorption studies. Cellulose.

[94] Wang N., Ouyang X.-K., Yang L.-Y., Omer A.M. (2017). Fabrication of a Magnetic Cellulose Nanocrystal/Metal–Organic Framework Composite for Removal of Pb(II) from Water. ACS Sustain. Chem. Eng..

[95] Ma X., Lou Y., Chen X.-B., Shi Z., Xu Y. (2019). Multifunctional flexible composite aerogels constructed through in-situ growth of metal-organic framework nanoparticles on bacterial cellulose. Chem. Eng. J..

[96] Ma S., Zhang M., Nie J., Tan J., Song S., Luo Y. (2019). Lightweight and porous cellulose-based foams with high loadings of zeolitic imidazolate frameworks-8 for adsorption applications. Carbohydr. Polym..

[97] Bo S., Ren W., Lei C., Xie Y., Cai Y., Wang S., Gao J., Ni Q., Yao J. (2018). Flexible and porous cellulose aerogels/zeolitic imidazolate framework (ZIF-8) hybrids for adsorption removal of Cr(IV) from water. J. Solid State Chem..

[98] Bahmani E., Seyyed Zonouzi H., Koushkbaghi S., Keyvani Hafshejani F., Fassadi Chimeh A., Irani M. (2020). Electrospun polyacrylonitrile/cellulose acetate/MIL-125/TiO_2_ composite nanofibers as an efficient photocatalyst and anticancer drug delivery system. Cellulose.

[99] Zhu H., Yang X., Cranston E.D., Zhu S. (2016). Flexible and Porous Nanocellulose Aerogels with High Loadings of Metal-Organic-Framework Particles for Separations Applications. Adv. Mater..

[100] Lei C., Gao J., Ren W., Xie Y., Abdalkarim S.Y.H., Wang S., Ni Q., Yao J. (2019). Fabrication of metal-organic frameworks@cellulose aerogels composite materials for removal of heavy metal ions in water. Carbohydr. Polym..

[101] Li J., Tan S., Xu Z. (2020). Anisotropic Nanocellulose Aerogel Loaded with Modified UiO-66 as Efficient Adsorbent for Heavy Metal Ions Removal. Nanomaterials.

[102] Hashem T., Ibrahim A.H., Wöll C., Alkordi M.H. (2019). Grafting Zirconium-Based Metal–Organic Framework UiO-66-NH_2_ Nanoparticles on Cellulose Fibers for the Removal of Cr(VI) Ions and Methyl Orange from Water. ACS Appl. Nano Mater..

[103] Gu Y., Wang Y., Li H., Qin W., Zhang H., Wang G., Zhang Y., Zhao H. (2020). Fabrication of hierarchically porous NH_2_-MIL-53/wood-carbon hybrid membrane for highly effective and selective sequestration of Pb^2+^. Chem. Eng. J..

[104] Yang W., Wang J., Yang Q., Pei H., Hu N., Suo Y., Li Z., Zhang D., Wang J. (2018). Facile fabrication of robust MOF membranes on cloth via a CMC macromolecule bridge for highly efficient Pb(II) removal. Chem. Eng. J..

[105] Liu J., Hao D., Sun H., Li Y., Han J., Fu B., Zhou J. (2021). Integration of MIL-101-NH_2_ into Cellulosic Foams for Efficient Cr(VI) Reduction under Visible Light. Ind. Eng. Chem. Res..

[106] Abdelhamid H.N., Mathew A. (2022). Cellulose-Metal Organic Frameworks (CelloMOFs) Hybrid Materials and their Multifaceted Applications: A Review. Coord. Chem. Rev..

[107] Baruah J., Chaliha C., Kalita E., Nath B.K., Field R.A., Deb P. (2020). Modelling and optimization of factors influencing adsorptive performance of agrowaste-derived Nanocellulose Iron Oxide Nanobiocomposites during remediation of Arsenic contaminated groundwater. Int. J. Biol. Macromol..

[108] Dong F., Xu X., Shaghaleh H., Guo J., Guo L., Qian Y., Liu H., Wang S. (2020). Factors influencing the morphology and adsorption performance of cellulose nanocrystal/iron oxide nanorod composites for the removal of arsenic during water treatment. Int. J. Biol. Macromol..

[109] Chen X., Cui J., Xu X., Sun B., Zhang L., Dong W., Chen C., Sun D. (2020). Bacterial cellulose/attapulgite magnetic composites as an efficient adsorbent for heavy metal ions and dye treatment. Carbohydr. Polym..

[110] Haniffa M.A.C.M., Munawar K., Chee C.Y., Pramanik S., Halilu A., Illias H.A., Rizwan M., Senthilnithy R., Mahanama K.R.R., Tripathy A. (2021). Cellulose supported magnetic nanohybrids: Synthesis, physicomagnetic properties and biomedical applications—A review. Carbohydr. Polym..

[111] Onwumere J., Pia̧tek J., Budnyak T., Chen J., Budnyk S., Karim Z., Thersleff T., Kuśtrowski P., Mathew A.P., Slabon A. (2020). CelluPhot: Hybrid Cellulose−Bismuth Oxybromide Membrane for Pollutant Removal. ACS Appl. Mater. Interfaces.

[112] Khan S.B., Alamry K.A., Marwani H.M., Asiri A.M., Rahman M.M. (2013). Synthesis and environmental applications of cellulose/ZrO_2_ nanohybrid as a selective adsorbent for nickel ion. Compos. Part B Eng..

[113] Brown A., Bozman M., Hickman T., Hossain M.I., Glover T.G., West K.N., West C.W. (2019). Superhydrophobic Functionalization of Cotton Fabric via Reactive Dye Chemistry and a Thiol-ene Click Reaction. Ind. Eng. Chem. Res..

[114] He X., Kim H., Dong T.G., Gates I., Lu Q. (2022). Green synthesis of Ag/lignin nanoparticle-loaded cellulose aerogel for catalytic degradation and antimicrobial applications. Cellulose.

[115] Soliman A.I.A., Díaz Baca J.A., Fatehi P. (2023). One-pot synthesis of magnetic cellulose nanocrystal and its post-functionalization for doxycycline adsorption. Carbohydr. Polym..

[116] Thomas M., Natarajan T.S., Sheikh M.U.D., Bano M., Khan F. (2017). Self-organized graphene oxide and TiO_2_ nanoparticles incorporated alginate/carboxymethyl cellulose nanocomposites with efficient photocatalytic activity under direct sunlight. J. Photochem. Photobiol. A Chem..

[117] Sun X., Danumah C., Liu Y., Boluk Y. (2012). Flocculation of bacteria by depletion interactions due to rod-shaped cellulose nanocrystals. Chem. Eng. J..

[118] Eyley S., Vandamme D., Lama S., Van den Mooter G., Muylaert K., Thielemans W. (2015). CO_2_ controlled flocculation of microalgae using pH responsive cellulose nanocrystals. Nanoscale.

[119] Pei A., Butchosa N., Berglund L.A., Zhou Q. (2013). Surface quaternized cellulose nanofibrils with high water absorbency and adsorption capacity for anionic dyes. Soft Matter.

[120] Sehaqui H., Mautner A., Perez de Larraya U., Pfenninger N., Tingaut P., Zimmermann T. (2016). Cationic cellulose nanofibers from waste pulp residues and their nitrate, fluoride, sulphate and phosphate adsorption properties. Carbohydr. Polym..

[121] Yao G., Zhu X., Wang M., Qiu Z., Zhang T., Qiu F. (2021). Controlled Fabrication of the Biomass Cellulose–CeO_2_ Nanocomposite Membrane as Efficient and Recyclable Adsorbents for Fluoride Removal. Ind. Eng. Chem. Res..

[122] Yu X., Tong S., Ge M., Zuo J. (2013). Removal of fluoride from drinking water by cellulose@hydroxyapatite nanocomposites. Carbohydr. Polym..

[123] Chen Y., Yang G., Liu B., Kong H., Xiong Z., Guo L., Wei G. (2022). Biomineralization of ZrO_2_ nanoparticles on graphene oxide-supported peptide/cellulose binary nanofibrous membranes for high-performance removal of fluoride ions. Chem. Eng. J..

[124] Darabitabar F., Yavari V., Hedayati A., Zakeri M., Yousefi H. (2020). Novel cellulose nanofiber aerogel for aquaculture wastewater treatment. Environ. Technol. Innov..

[125] Robledo-Peralta A., Torres-Castañón L.A., Rodríguez-Beltrán R.I., Reynoso-Cuevas L. (2022). Lignocellulosic Biomass as Sorbent for Fluoride Removal in Drinking Water. Polymers.

[126] Raval P., Thomas N., Hamdouna L., Delevoye L., Lafon O., Manjunatha Reddy G.N. (2023). Boron Adsorption Kinetics of Microcrystalline Cellulose and Polymer Resin. Langmuir.

[127] Fang S., Guan K., Mai Z., Zhou S., Song Q., Li Z., Xu P., Hu M., Chiao Y.-H., Zhang P. (2023). Complexation of cellulose nanocrystals and amine monomer for improved interfacial polymerization of nanofiltration membrane. J. Memb. Sci..

[128] Wang S., Chen X., Li Z., Zeng W., Meng D., Wang Y., Xiao Z., Wang H., Liang D., Xie Y. (2022). Click chemistry-induced selective adsorption of cationic and anionic dyes using functionalized cellulose methacrylate hydrogels. Cellulose.

[129] Qiu C., Li Y., Liu H., Wang X., Hu S., Qi H. (2023). A novel crosslinking strategy on functional cellulose-based aerogel for effective and selective removal of dye. Chem. Eng. J..

[130] Goetz L.A., Naseri N., Nair S.S., Karim Z., Mathew A.P. (2018). All cellulose electrospun water purification membranes nanotextured using cellulose nanocrystals. Cellulose.

[131] Yu S., Liu M., Ma M., Qi M., Lü Z., Gao C. (2010). Impacts of membrane properties on reactive dye removal from dye/salt mixtures by asymmetric cellulose acetate and composite polyamide nanofiltration membranes. J. Memb. Sci..

[132] Zhou C., Wu Q., Lei T., Negulescu I.I. (2014). Adsorption kinetic and equilibrium studies for methylene blue dye by partially hydrolyzed polyacrylamide/cellulose nanocrystal nanocomposite hydrogels. Chem. Eng. J..

[133] Li Y., Xiao H., Pan Y., Wang L. (2018). Novel Composite Adsorbent Consisting of Dissolved Cellulose Fiber/Microfibrillated Cellulose for Dye Removal from Aqueous Solution. ACS Sustain. Chem. Eng..

[134] Kaushik M., Moores A. (2016). Review: Nanocelluloses as versatile supports for metal nanoparticles and their applications in catalysis. Green Chem..

[135] Xu Y., Zhang Z., Cui Z., Luo L., Lin P., Xie M., Zhang Q., Sa B., Wen C. (2024). Enhanced interfacial interactions and enriched active sites in self-assembly amino-functionalized bacterial cellulose/MXene composite for wastewater treatment. Chem. Eng. J..

[136] Zhang W., Ji X.-X., Ma M.-G. (2023). Emerging MXene/cellulose composites: Design strategies and diverse applications. Chem. Eng. J..

[137] Almeida da Silva T.C., Marchiori L., Oliveira Mattos B., Ullah S., Barud H.d.S., Romano Domeneguetti R., Rojas-Mantilla H.D., Boldrin Zanoni M.V., Rodrigues-Filho U.P., Ferreira-Neto E.P. (2023). Designing Highly Photoactive Hybrid Aerogels for In-Flow Photocatalytic Contaminant Removal Using Silica-Coated Bacterial Nanocellulose Supports. ACS Appl. Mater. Interfaces.

[138] Zou M., Tan C., Yuan Z., Zhang L., Wu M., Hu J., Ma Z., Zhou H. (2024). In situ generated CdS on mixed cellulose membrane matrix as an easily recyclable photocatalyst for mineral flotation wastewater treatment. Cellulose.

[139] Wang Z., Zhang X.-F., Shu L., Yao J. (2023). Copper sulfide integrated functional cellulose hydrogel for efficient solar water purification. Carbohydr. Polym..

[140] Zhu L., Zong L., Wu X., Li M., Wang H., You J., Li C. (2018). Shapeable Fibrous Aerogels of Metal–Organic-Frameworks Templated with Nanocellulose for Rapid and Large-Capacity Adsorption. ACS Nano.

[141] Vatanpour V., Yuksekdag A., Ağtaş M., Mehrabi M., Salehi E., Castro-Muñoz R., Koyuncu I. (2023). Zeolitic imidazolate framework (ZIF-8) modified cellulose acetate NF membranes for potential water treatment application. Carbohydr. Polym..

[142] Lee K., Jeon Y., Kwon G., Lee S., Ko Y., Park J., Kim J., You J. (2024). Multiporous ZIF-8 carbon/cellulose composite beads: Highly efficient and scalable adsorbents for water treatment. Carbohydr. Polym..

[143] Uchiyama K., Asamoto H., Minamisawa H., Yamada K. (2023). Quaternization of Porous Cellulose Beads and Their Use for Removal of Humic Acid from Aqueous Medium. Physchem.

[144] Vandamme D., Eyley S., Van den Mooter G., Muylaert K., Thielemans W. (2015). Highly charged cellulose-based nanocrystals as flocculants for harvesting *Chlorella vulgaris*. Bioresour. Technol..

[145] Mousa H.M., Fahmy H.S., Ali G.A.M., Abdelhamid H.N., Ateia M. (2022). Membranes for Oil/Water Separation: A Review. Adv. Mater. Interfaces.

[146] Yin Z., Li Z., Deng Y., Xue M., Chen Y., Ou J., Xie Y., Luo Y., Xie C., Hong Z. (2023). Multifunctional CeO_2_-coated pulp/cellulose nanofibers (CNFs) membrane for wastewater treatment: Effective oil/water separation, organic contaminants photodegradation, and anti-bioadhesion activity. Ind. Crop. Prod..

[147] Abbas S.M., Al-Jubouri S.M. (2024). High performance and antifouling zeolite@polyethersulfone/cellulose acetate asymmetric membrane for efficient separation of oily wastewater. J. Environ. Chem. Eng..

[148] Elgueta E., Becerra Y., Martínez A., Pereira M., Carrillo-Varela I., Sanhueza F., Nuñez D., Rivas B.L. (2023). Adsorbents Derived from Xylan Hemicellulose with Removal Properties of Pollutant Metals. Chin. J. Polym. Sci..

[149] Kausar A., Zohra S.T., Ijaz S., Iqbal M., Iqbal J., Bibi I., Nouren S., El Messaoudi N., Nazir A. (2023). Cellulose-based materials and their adsorptive removal efficiency for dyes: A review. Int. J. Biol. Macromol..

[150] Morsi R.E., Corticelli F., Morandi V., Gentili D., Cavallini M., Figoli A., Russo F., Galiano F., Aluigi A., Ventura B. (2023). Influence of the Fabrication Conditions on the Physical Properties and Water Treatment Efficiency of Cellulose Acetate Porous Membranes. Water.

[151] Hazarika T., Kakati B., Pal D., Saikia R., Rawal A., Mahanta M.K., Biswas S. (2024). Role of plasma process gas on permeate flux augmentation of cellulose nitrate membrane for mud water treatment. Sci. Rep..

[152] Namasivayam S.K.R., Prakash P., Babu V., Paul E.J., Bharani R.S.A., Kumar J.A., Kavisri M., Moovendhan M. (2023). Aquatic biomass cellulose fabrication into cellulose nanocomposite and its application in water purification. J. Clean. Prod..

[153] Spinu M., Dos Santos N., Le Moigne N., Navard P. (2011). How does the never-dried state influence the swelling and dissolution of cellulose fibres in aqueous solvent?. Cellulose.

[154] Zhang Z., Ahmed A.I.S., Malik M.Z., Ali N., Khan A., Ali F., Hassan M.O., Mohamed B.A., Zdarta J., Bilal M. (2023). Cellulose/inorganic nanoparticles-based nano-biocomposite for abatement of water and wastewater pollutants. Chemosphere.

[155] Azimi B., Sepahvand S., Ismaeilimoghadam S., Kargarzadeh H., Ashori A., Jonoobi M., Danti S. (2024). Application of Cellulose-Based Materials as Water Purification Filters; A State-of-the-Art Review. J. Polym. Environ..

